# Targeting human mitochondrial NAD(P)^+^-dependent malic enzyme (ME2) impairs energy metabolism and redox state and exhibits antileukemic activity in acute myeloid leukemia

**DOI:** 10.1007/s13402-023-00812-x

**Published:** 2023-04-20

**Authors:** Kun-Chi Chen, I-Hsin Hsiao, Yu-Nan Huang, Yu-Tung Chou, Yi-Chun Lin, Ju-Yi Hsieh, Yung-Lung Chang, Kang-Hsi Wu, Guang-Yaw Liu, Hui-Chih Hung

**Affiliations:** 1 Department of Life Sciences, National Chung Hsing University, Taichung, Taiwan; 2 Ph.D. Program in Tissue Engineering and Regenerative Medicine, National Chung Hsing University, Taichung, Taiwan; 3 Department of Biochemistry, National Defense Medical Center, Taipei, Taiwan; 4 School of Medicine, Chung Shan Medical University, Taichung, Taiwan; 5 Department Pediatrics, Chung Shan Medical University Hospital, Taichung, Taiwan; 6 Institute of Medicine, College of Medicine, Chung Shan Medical University, Taichung, Taiwan; 7 Institute of Genomics and Bioinformatics, National Chung Hsing University, Taichung, Taiwan; 8 iEGG & Animal Biotechnology Center, National Chung Hsing University, Taichung, Taiwan

**Keywords:** ME2 silencing, Pyruvate metabolism, ROS homeostasis, Apoptosis, Cellular respiration

## Abstract

Acute myeloid leukemia (AML) is a fast-growing and highly fatal blood cancer, and recent research has shown that targeting metabolism may be a promising therapeutic approach for treating AML. One promising target is the human mitochondrial NAD(P)^+^-dependent malic enzyme (ME2), which is involved in the production of pyruvate and NAD(P)H and the regulation of the NAD^+^/NADH redox balance. Inhibition of ME2 via silencing ME2 or utilizing its allosteric inhibitor disodium embonate (Na_2_EA) causes a decrease in pyruvate and NADH, leading to a decrease in producing ATP via cellular respiration and oxidative phosphorylation. ME2 inhibition also decreases NADPH levels, resulting in an increase in reactive oxygen species (ROS) and oxidative stress, which ultimately leads to cellular apoptosis. Additionally, ME2 inhibition reduces pyruvate metabolism and the biosynthetic pathway. ME2 silencing inhibits the growth of xenotransplanted human AML cells, and the allosteric ME2 inhibitor Na_2_EA demonstrates antileukemic activity against immune-deficient mice with disseminated AML. Both of these effects are a result of impaired energy metabolism in mitochondria. These findings suggest that the targeting ME2 may be an effective strategy for treating AML. Overall, ME2 plays an essential role in energy metabolism of AML cells, and its inhibition may offer a promising approach for AML treatment.

**Supplementary Information:**

The online version contains supplementary material available at 10.1007/s13402-023-00812-x.

## Introduction

Acute myeloid leukemia (AML) is a malignant disease characterized by the fast proliferation of aberrant cells that cluster in the bone marrow and blood and disrupt normal blood cells [1, 2]. Certain cytogenetic and molecular abnormalities have been identified in AML patients, and these genetic variations or mutations that occur in AML patients are currently used to guide therapy and predict the prognosis of an individual with AML [3, 4]. The deregulation of tyrosine kinases (TKs) in AML may contribute to the development of leukemia by promoting cell proliferation and survival [5]. It has been discovered that AML cells are highly dependent on oxidative metabolism [6–8] and that chemoresistant AML cells exhibit greater oxidative phosphorylation and are more sensitive to inhibiting cell respiration [9].

Malic enzymes are oxidative decarboxylases that catalyze the oxidative decarboxylation of L-malate to produce CO_2_ and pyruvate while concurrently reducing NAD(P)^+^ to NAD(P)H [10, 11]. The two predominant isoforms in cells are the cytosolic NADP^+^-dependent malic enzyme (ME1) and the mitochondrial NAD(P)^+^-dependent malic enzyme (ME2). ME2 possesses dual cofactor specificity [12–14], allowing it to generate either NADH or NADPH, which are used in different metabolic processes in the cell. NADH is used as a reducing equivalent to produce ATP through oxidative phosphorylation, whereas NADPH is employed as an antioxidant to reduce the ROS level in the cell. Fumarate, an intermediate of the tricarboxylic acid cycle (TCA cycle), is an allosteric activator of ME2 [15], which has been considered an oncometabolite [16], whereas ATP may act as an active site and exo-site inhibitor of ME2 [14, 17, 18]. It indicates that the flux of the TCA cycle and the energy status are tightly regulating the activity of ME2 [19]. Both ME1 and ME2 have been identified as targets of p53, a tumor suppressor protein that can restrict their expression via transcriptional repression. Downregulation of ME1 and ME2 can result in p53 activation through different pathways [20].

Altered metabolism is a hallmark of tumor growth [21], and cancer cells rely on multiple unique metabolic pathways to support their growth and survival. These pathways include aerobic glycolysis (the Warburg effect), glutamine and acetate addictions, and ammonia recycling [22–26]. Metabolic reprogramming is a typical survival and growth mechanism for cancer cells, as they need to adapt to their environment to ensure their survival and proliferation [21, 25]. ME2 plays a critical role in mediating metabolic alterations in cancer cells [27]. For example, ME2 expression has been shown to increase significantly during the progression of cutaneous melanoma, in part due to its regulation of the AMPK pathway, which is related to the energy required for cell mobility [27]. A ME2-specific inhibitor embonic acid (EA) has been shown to inhibit lung cancer cell growth and induce cellular senescence by inhibiting ME2 [28]. An allosteric activator of ME2, fumarate, functions as a signaling molecule to regulate mitochondrial biogenesis via the metabolite-sensing pathway [16].

It is believed that AML cells are highly dependent on glutamine for their survival [29]. The deficiency of pyruvate from glycolysis is replenished by glutaminolysis for mitochondrial anaplerosis, which involves ME2 in the production of sufficient energy for rapid cell proliferation [10, 30]. ME2 recycles malate into pyruvate in mitochondria, regulating the flux of the TCA cycle to meet the requirements for cellular energy, carbon sources, and reducing equivalents [19, 25, 31, 32]. AML is particularly dependent on mitochondrial function and is susceptible to the fumarate-ME2 axis [16]. Here, we investigate the role of ME2 in AML metabolic adaptations. We found that ME2 depletion reduces mitochondrial metabolism, including pyruvate metabolism, cellular respiration and oxidative phosphorylation. The effect of a ME2-specific inhibitor was comparable to that of ME2 depletion in AML. This study revealed the role of ME2 in AML cell energy metabolism and ROS homeostasis. As a result, we suggested that targeting ME2 as a therapeutic approach for AML treatment.

## Results

### ME2 mRNA and protein levels were elevated in acute myeloid leukemia (AML)

Using the microarray dataset GDS3057 [33] from the NCBI Gene Expression Omnibus (GEO) database, we analyzed the expression of ME2. The samples were categorized as either normal/AML in bone marrow or peripheral blood (Figure S1A). AML had higher ME2 mRNA levels than normal bone marrow, but not peripheral blood (Figure S1A). The expression of ME2 protein levels was then analyzed in several AML cell lines (Figure S1B), and HL-60 (M3), THP-1 (M5), MV4-11 (M4), and OCI-AML-2 (M4) were found to strongly express ME2. The ME2 gene was silenced in HL-60, THP-1, and MV4-11 cells using two shRNA sequences (shME2-1 and shME2-2), and the knockdown efficiency was confirmed using western blotting (Figures S1C, S1D, and S1E for HL-60, THP-1, and MV4-11 cells, respectively) and flow cytometry (Figures S1F, S1G, and S1H for shCtrl, shME2-1, and shME2-2 in HL-60 cells, respectively).

### ME2 silencing results in decreased ATP and NADPH levels, which correlates with increased ROS and cellular apoptosis

**Fig. 1:**
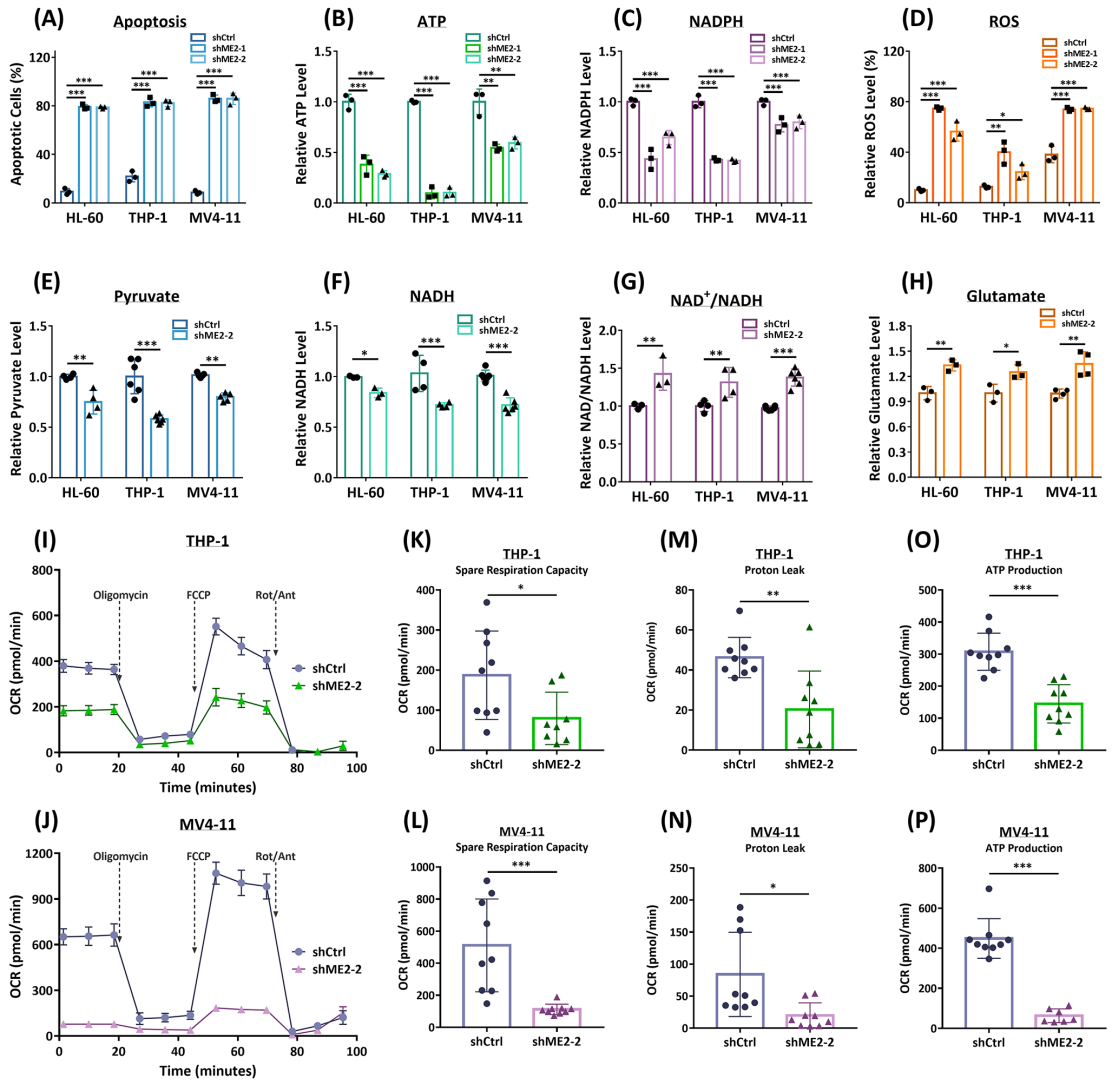
Metabolic changes in ME2-silenced AML cells. Three AML cell lines, HL-60, THP-1, and MV4-11, were utilized to examine the metabolic changes that occurred when ME2 was silenced. **(A)** The relative changes in cellular apoptosis (%). **(B)** The relative changes in ATP level were measured by fluorescence-based flow cytometry. **(C)** The relative changes in NADPH levels. **(D)** The relative changes in ROS levels (%). **(E)** The relative changes in pyruvate levels. **(F)** The relative changes in NADH levels. **(G)** The relative changes in NAD^+^/NADH ratio. **(H)** The relative changes in glutamate levels. **(I)** and **(J)** The oxygen consumption rate in ME2-control (shCtrl) and ME2-knockdown (shME2) THP-1 and MV4-11 cells, respectively. **(K)** and **(L)** The spare respiration capacity of THP-1 and MV4-11 cells, respectively. **(M)** and **(N)** The proton leak of THP-1 and MV4-11 cells, respectively. **(O)** and **(P)** ATP production in THP-1 and MV4-11 cells, respectively. The bar graphs illustrate the fold change in the levels of these metabolites or OCR-derived factors (n = 3 or more in each group, mean ± SD). *p < 0.05, **p < 0.01, and ***p < 0.001. *p* values were calculated using the Student’s *t*-test

ME2 was found to be expressed by multiple AML cells (Figure S1B), and the knockdown of ME2 significantly decreased the proliferation of HL-60, THP-1, and MV4-11 AML cell lines (Figures S2A, S2B, and S2C, respectively). Flow cytometry analysis utilizing both Annexin V conjugates and propidium iodide (PI) staining to detect apoptotic cells revealed that ME2 silencing resulted in up to 80% cellular apoptosis in three AML cell lines (Fig. 1A and S2D), indicating that ME2 is essential for AML cell growth.

Furthermore, ME2 silencing has a substantial impact on ATP production in AML cells. ME2-silenced HL-60, THP-1, and MV4-11 AML cell lines contained significantly less intracellular ATP than the control group (Fig. 1B), indicating that ME2 plays an essential role in AML energy metabolism.

ME2 converts L-malate to pyruvate simultaneously with NADPH production, and the knockdown of ME2 decreased NADPH production (Fig. 1C), resulting in high ROS content remaining in the cell (Fig. 1D). This increase in ROS may be the cause of cellular apoptosis in these AML cells. These findings demonstrate that ME2 is essential for the growth and production of ATP in AML cells, as the knockdown of ME2 results in decreased ATP and NADPH levels, increased ROS content, and cellular apoptosis.

### ME2 silencing decreases pyruvate and NADH levels, thereby disturbing energy metabolism

ME2 is involved in the production of pyruvate and NADH, which are directly involved in the production of cellular ATP via cellular respiration and oxidative phosphorylation. It is anticipated that the ME2-generated mitochondrial pyruvate will convert to acetyl-CoA before entering the TCA cycle and ultimately producing ATP via the respiration chain. In addition, the ratio of NAD^+^ to NADH is an important indicator of cellular energy metabolism. Therefore, the levels of pyruvate, NADH, and the NAD^+^/NADH ratio were determined in ME2-silenced HL-60, THP-1, and MV4-11 AML cells. The decrease in pyruvate and NADH levels (Fig. 1E, and 1 F, respectively), as well as the increase in the NAD^+^/NADH ratio (Fig. 1G) observed in ME2-silenced AML cells, suggests that ME2 plays a critical role in energy metabolism in AML cells. Furthermore, ME2 silencing decreased glutamate utilization in AML cells, as evidenced by a higher glutamate level in ME2-silenced HL-60, THP-1, and MV4-11 AML cells than in the control group of AML cells (Fig. 1H), indicating a positive role of ME2 in glutamate utilization of AML cells.

### ME2 silencing decreases cellular respiration and oxidative phosphorylation

The oxygen consumption rate (OCR) experiments were carried out using the Agilent Seahorse XF Analyzer to assess the effect of ME2 silencing on the respiration capacity and the amount of ATP produced by oxidative phosphorylation in AML cells. The OCR was significantly decreased in ME2-silenced HL-60, THP-1, and MV4-11 AML cells (Figures S3A, 1I, and 1J, respectively), as evidenced by the basal (Figures S3B, S3C, and S3D, respectively) and maximal respiration (Figures S3E, S3F, and S3G, respectively), as well as the spare respiration capacity (Figures S3H, 1 K, and 1 L, respectively), the mitochondrial proton leak (Figures S3I, 1 M, and 1 N, respectively), and the amount of ATP generated by mitochondrial electron transport (Figures S3J, 1O, and 1P, respectively).

The decreased OCR, including the mitochondrial proton leak and ATP produced by oxidative phosphorylation, as well as the decreased ATP, pyruvate, and NADH levels in ME2-silenced HL-60, THP-1, and MV4-11 AML cells (Fig. 1B and E, and 1 F, respectively), provided conclusive evidence that ME2 regulates energy metabolism through cellular respiration and oxidative phosphorylation.

### ME2 silencing inhibits numerous metabolic pathways that correspond to energy metabolism and biosynthetic pathways

**Fig. 2:**
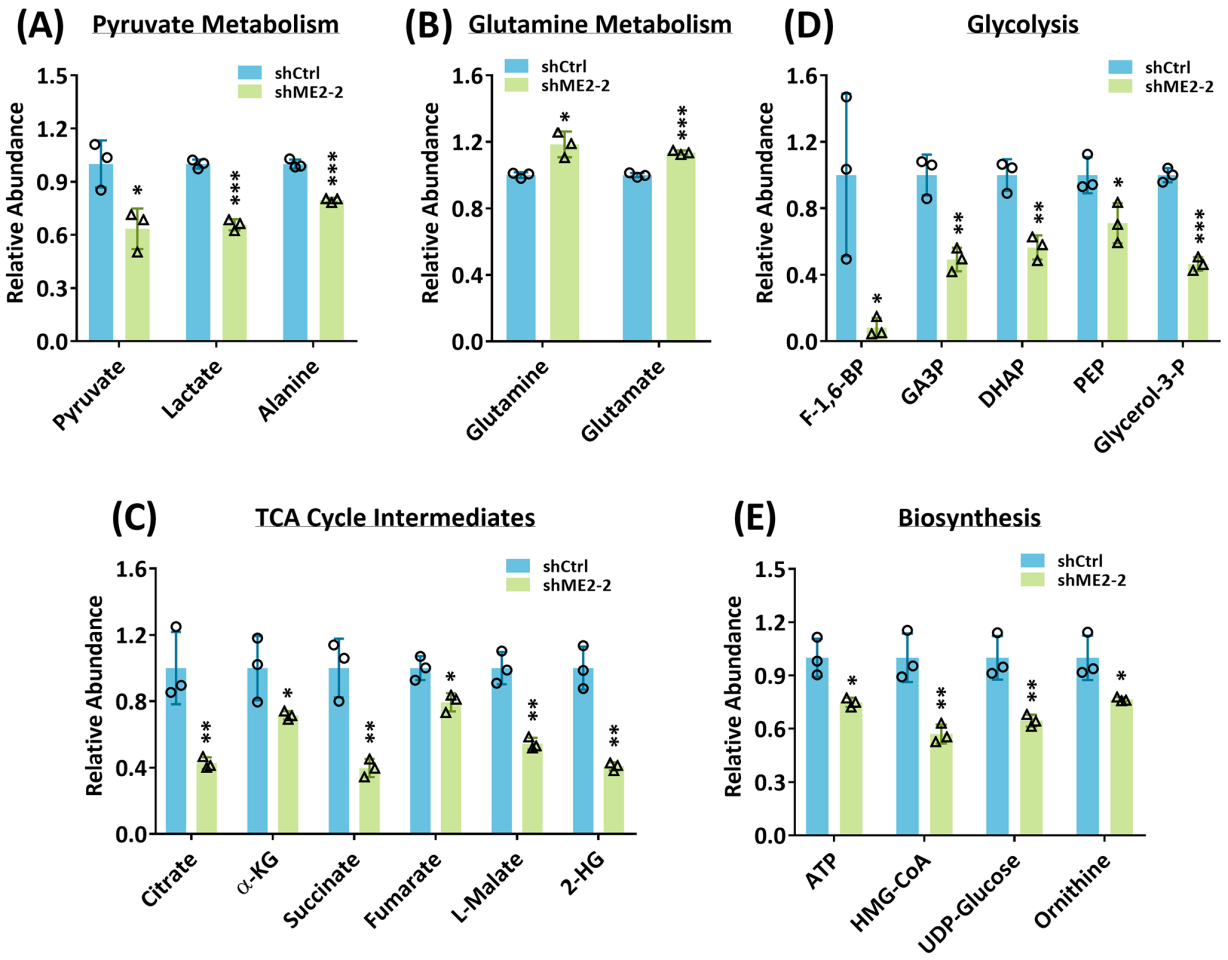
Changes in metabolic intermediate levels in ME2-silenced MV4-11 cells. The changes of metabolic intermediates in ME2-control (shCtrl) and ME2-knockdown (shME2) MV4-11 cells were analyzed using mass spectrometry. Intermediates are present in **(A)** pyruvate metabolism, **(B)** glutamine metabolism, **(C)** the TCA cycle, **(D)** glycolysis, and **(E)** the biosynthetic pathway

The results of a mass analysis experiment in which ME2 silencing was found to have several effects on cellular metabolism. ME2 silencing led to a decrease in pyruvate levels, which in turn caused a reduction in the abundance of lactate and alanine (Fig. 2A). This suggests that ME2 is involved in the conversion of pyruvate to lactate and alanine. Furthermore, ME2 silencing was also found to decrease the utilization of glutamine and glutamate (Fig. 2B), which may lead to a decrease in the levels of TCA-cycle intermediates (Fig. 2C). In addition, ME2 silencing led to a decrease in glycolysis (Fig. 2D) as well as the levels of the biosynthetic precursors HMG-CoA, UDP-glucose, and ornithine (Fig. 2E). These biosynthetic precursors were used to synthesize cholesterol, glycogen, and polyamine, all of which are essential for growth. Briefly, the mass analysis performed by silencing ME2 suggests that ME2 is involved in regulating glycolysis, pyruvate metabolism, the utilization of glutamine and glutamate, and the biosynthesis of these molecules.

### ME2 silencing inhibits the growth of xenotransplanted human AML cells

**Fig. 3:**
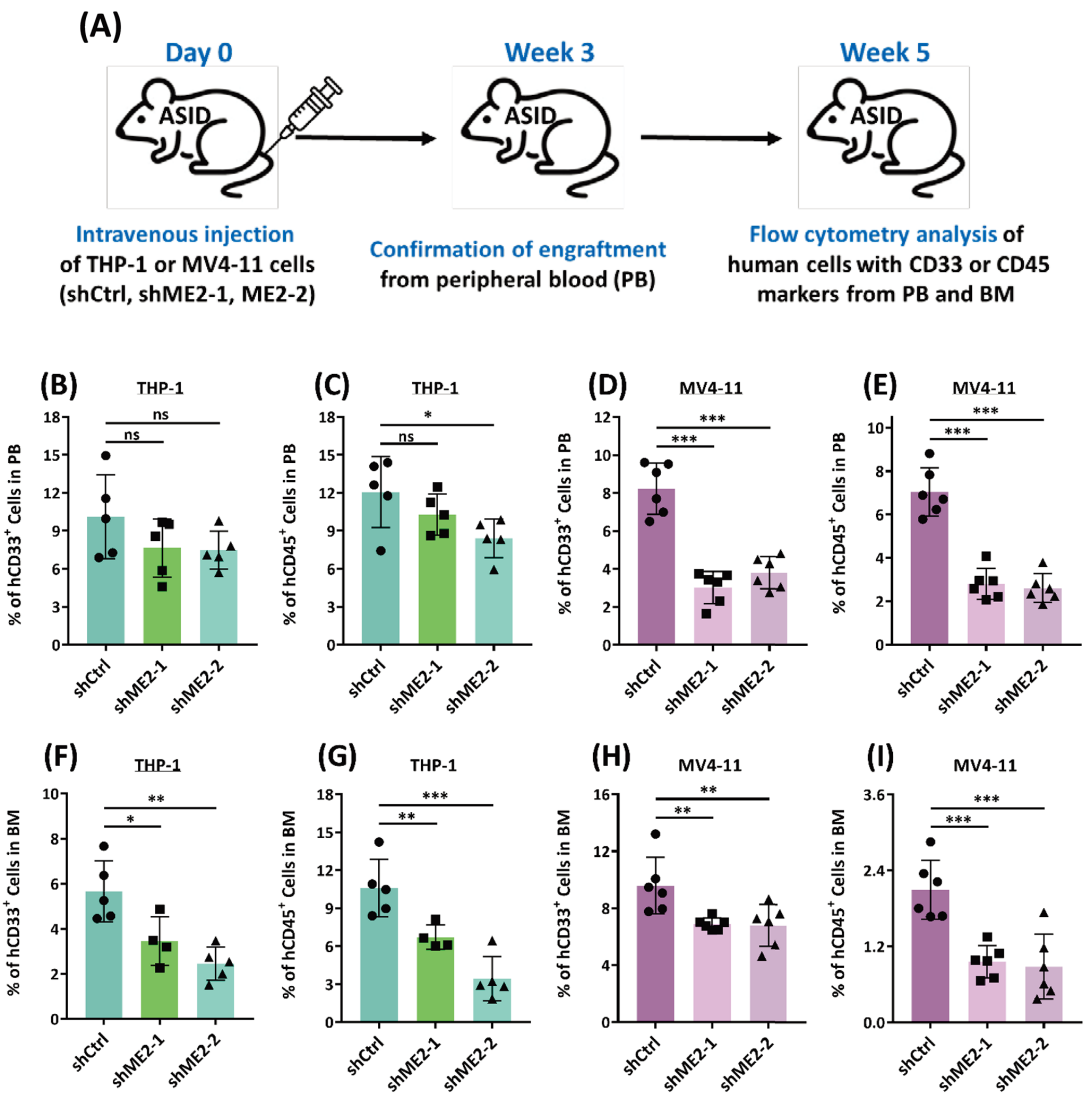
ME2-silenced cell line-derived xenograft model studies. **(A)** Schematic representation of ASID mice implanted with ME2-control (shCtrl) and ME2-knockdown (shME2) THP-1 and MV4-11 cells. **(B)** and **(C)** Percentage of CD33 + and CD45 + cells, respectively, in shME2-THP-1 cells in the peripheral blood (PB) of mice after five weeks. **(D)** and **(E)** Percentage of CD33 + and CD45 + cells, respectively, in shME2-MV4-11 cells in the peripheral blood (PB) of mice after five weeks. **(F)** and **(G)** Percentage of CD33 + and CD45 + cells, respectively, in shME2-THP-1 cells in the bone marrow (BM) of mice after five weeks. **(H)** and **(I)** Percentage of CD33 + and CD45 + cells, respectively, in shME2-MV4-11 cells in the bone marrow (BM) of mice after five weeks

AML cells express the myeloid differentiation antigen CD33 and the leukocyte common antigen CD45, which is highly expressed in leukemogenic AML cells and serves as a marker for all hematopoietic cells. Targeting CD33 or CD45 is the basis of current AML immunotherapy [34]. To further evaluate the role of ME2 in *vivo* development of leukemia, ME2-silenced THP-1 and MV4-11 AML cells as well as control cells were implanted into mice with advanced severe immunodeficiency (ASID); the percentage of CD33 + and CD45 + cells in the peripheral blood (PB) and bone marrow (BM) compartments were used to assess the burden of AML (Fig. 3A). After three weeks of xenotransplantation, the engraftment efficiency of THP-1 and MV4-11 AML cells was confirmed by monitoring CD33 + or CD45 + cells in the peripheral blood of shME2-THP-1 or shME2-MV4-11 mice (Figure S4).

The burden of AML in shME2-THP-1 and shME2-MV4-11 mice was determined five weeks after xenotransplantation by monitoring the proportional change of CD33 + and CD45 + cells in the peripheral blood and bone marrow (Figures S5 and S6, respectively). Monitoring AML cells in the PB of shME2-THP-1 mice revealed a decrease in CD45 + cells (Fig. 3C), but not CD33 + cells (Fig. 3B). In contrast, ME2 silencing significantly decreased the number of AML cells in shME2-MV4-11 mice by monitoring CD33 + and CD45 + cells in the peripheral blood (Fig. 3D and E). Monitoring CD33 + or CD45 + cells from the bone marrow, ME2 silencing in both shME2-THP-1 (Fig. 3F and G) and shME2-MV4-11 (Fig. 3H and I, respectively) mice resulted in a significant decrease of AML cells, which may be attributed to the impairment of ME2-associated energy metabolism and redox balance, suggesting a positive role for ME2 in the development of leukemia.

### The allosteric ME2 inhibitor embonic acid (EA) has a significant effect on mitochondrial metabolism and ROS homeostasis in AML cells

Our laboratory has identified an allosteric ME2 inhibitor, embonic acid (EA), which inhibits ME2 activity specifically [28]. In this study, we use the disodium salt of EA (Na_2_EA) to determine its inhibitory effect on three AML cell lines and xenotransplanted mice.

**Fig. 4:**
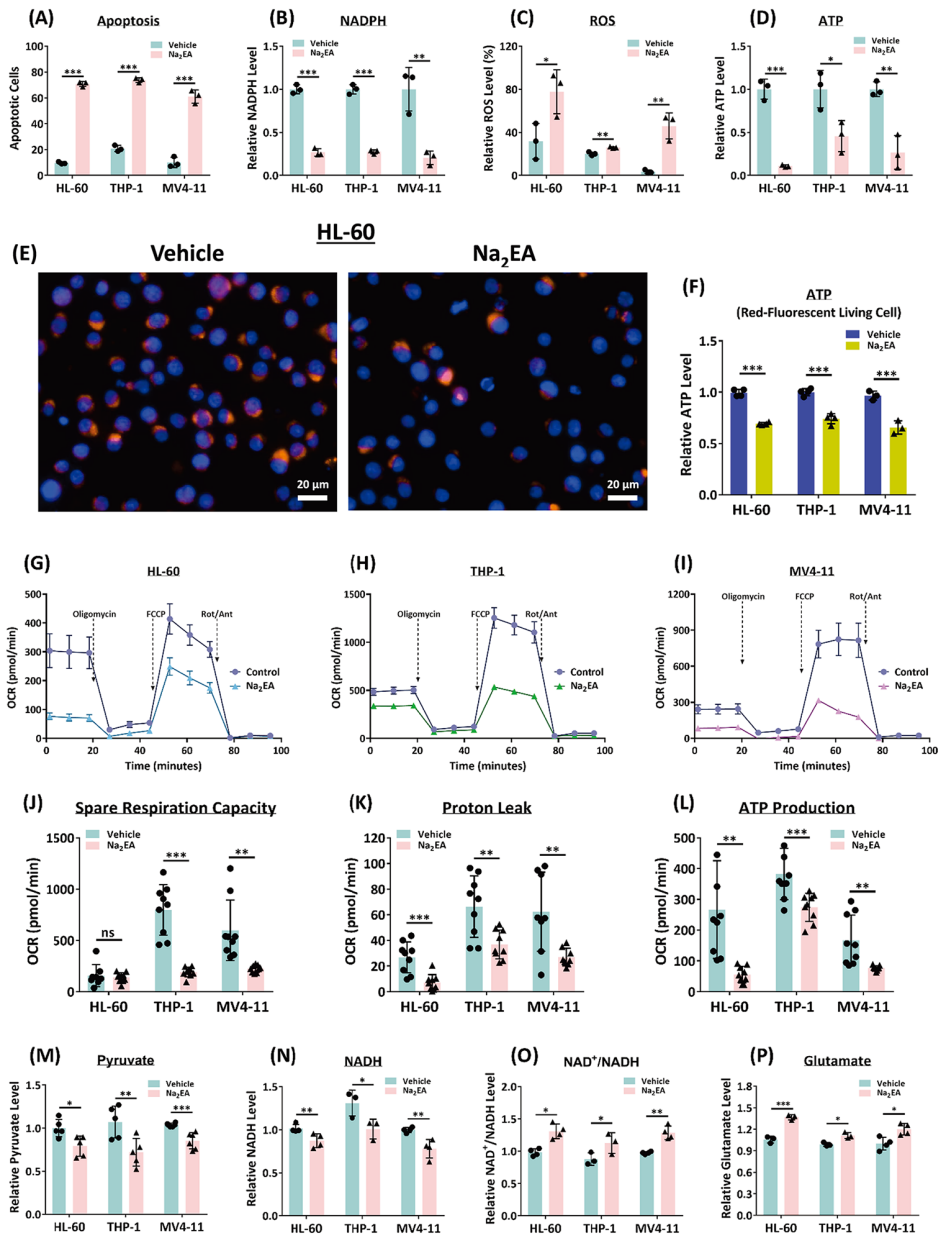
Metabolic changes in Na_2_EA-treated AML cells. Three AML cell lines, HL-60, THP-1, and MV4-11, were treated with the ME2 inhibitor Na_2_EA to examine the metabolic changes. **(A)** The relative change in cellular apoptosis (%). **(B)** The fold changes in NADPH levels. **(C)** The relative changes in ROS levels (%). **(D)** The relative changes in ATP levels. **(E)** Living cell red fluorescent imaging for ATP. **(F)** ATP levels quantified by Live cell fluorescent imaging. **(G)**, **(H)** and **(I)** The oxygen consumption rate in ME2-control (Vehicle) and ME2-inhibited (treated with Na_2_EA) HL-60, THP-1, and MV4-11 cells, respectively. **(J), (K), and (L)** The spare respiration capacity, proton leak, and the ATP production of HL-60, THP-1, and MV4-11 cells. **(M)** The relative changes in pyruvate levels. **(N)** The relative changes in NADH levels. **(O)** The relative changes in NAD^+^/NADH ratio. **(P)** The relative changes in glutamate levels. The bar graphs illustrate the fold change in the level of these metabolic intermediates after 48 h (n = 3 or more in each group, mean ± SD). *p < 0.05, **p < 0.01, and ***p < 0.001. *p* values were calculated using the Student’s *t*-test

The IC_50_ values of the cell viability of HL-60, THP-1, and MV4-11 AML cell lines are approximately 120 µM when treated with Na_2_EA (Figure S7A, S7B, and S7C, respectively), and therefore the AML cell lines were treated with 150 µM of Na_2_EA for the subsequent experiments. Flow cytometry analysis to detect apoptosis revealed that Na_2_EA induced 60–75% cellular apoptosis in three AML cell lines (Fig. 4A and S7D-S7I); ME2 inhibition by Na_2_EA treatment also decreased NADPH production (Fig. 4B), which resulted in ROS accumulation in the cell (Fig. 4C), indicating that ME2 inhibition disrupted ROS homeostasis and was therefore detrimental to AML cell growth.

Treatment with Na_2_EA significantly decreased ATP production in HL-60, THP-1, and MV4-11 cells (Fig. 4D); living cell images (Fig. 4E and S9) and the counting of ATP-containing cells (Fig. 4F) also demonstrated lower ATP content in Na_2_EA-treated HL-60, THP-1, and MV4-11 AML cells; the OCR was reduced in Na_2_EA-treated AML cells (Fig. 4G and I), as were the basal (Figures S8A-S8C) and maximal respiration (Figures S8D-S8F), as well as the spare respiration capacity (Fig. 4J), the mitochondrial proton leak (Fig. 4K), and the amount of ATP generated by mitochondrial electron transport chain (Fig. 4L). These findings indicate that ME2 inhibition by Na_2_EA disrupts ATP homeostasis in AML cells.

Similar to ME2-silenced cells, treatment with Na_2_EA inhibited ME2 activity in HL-60, THP-1, and MV4-11 AML cell lines, resulting in a decrease in pyruvate and NADH levels and an increase in the NAD^+^/NADH ratio (Fig. 4 M, 4 N and 4O, respectively), indicating that ME2 inhibition is detrimental to energy metabolism, which corresponds cellular respiration and ATP synthesis. Treatment with Na_2_EA inhibited glutamate utilization in HL-60, THP-1, and MV4-11 AML cells, resulting in glutamate accumulation within the cells (Fig. 4P).

### Na_2_EA treatment significantly impaired energy metabolism

**Fig. 5:**
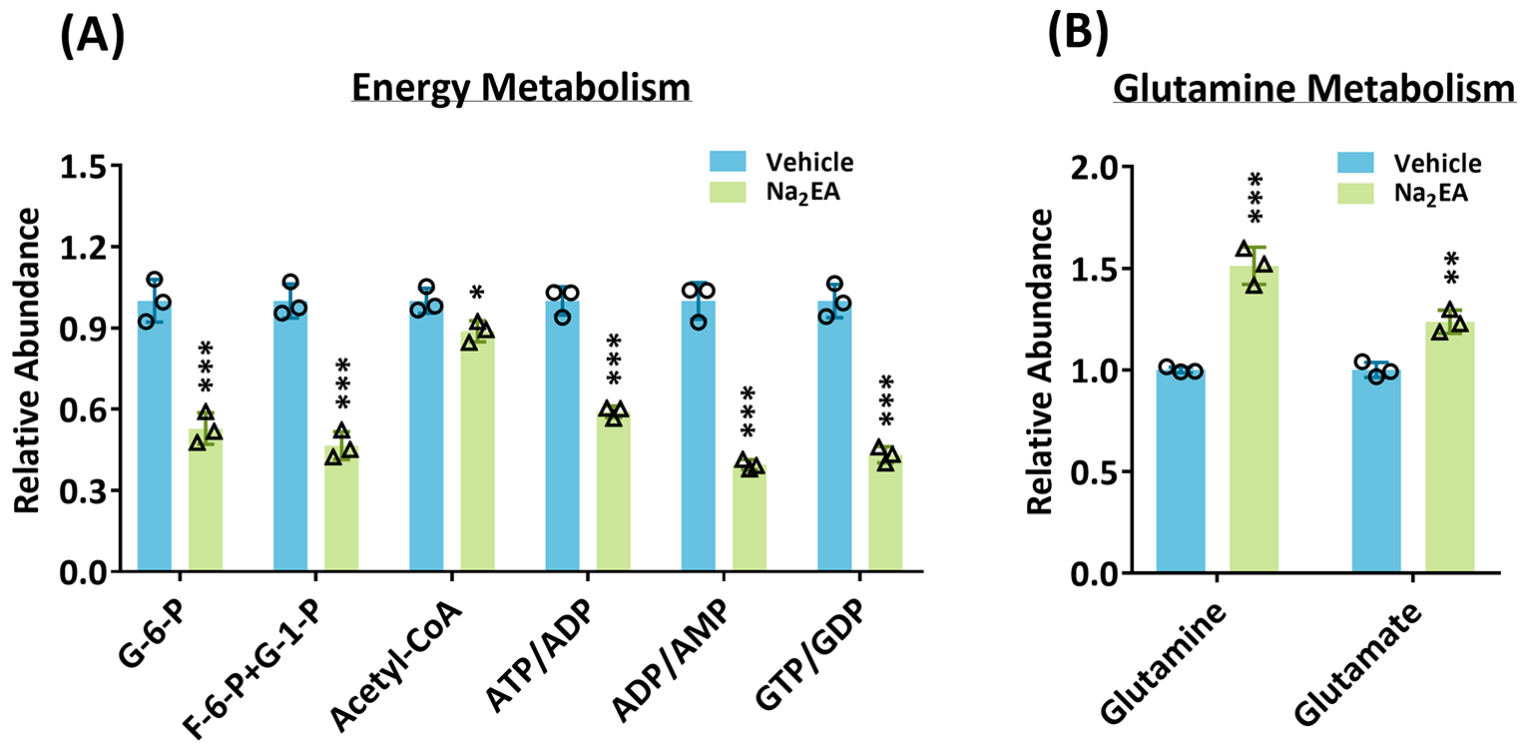
Changes in metabolite levels in Na_2_EA-treated HL-60 cells. The changes in metabolic intermediates in HL-60 cells were analyzed using mass spectrometry. **(A)** Intermediates are involved in **(A)** energy metabolism, and **(B)** glutamine metabolism

The inhibition of ME2 using Na_2_EA was found to significantly reduce the levels of glycolytic intermediates glucose-6-phosphate (G-6-P) and fructose-6-phosphate (F-6-P), as well as glycogenolysis intermediate glucose-1-phosphate (G-1-P) (Fig. 5A). This indicates that ME2 plays a role in the regulation of glycolysis and glycogenolysis, which are involved in energy metabolism. Furthermore, the inhibition of ME2 by Na_2_EA also led to the suppression of glutamine and glutamate utilization, which was demonstrated by elevated levels of glutamine and glutamate (Fig. 5B). As a result of the inhibition of glycolysis and glycogenolysis, the levels of energy indicators such as ATP/ADP, ADP/AMP, and GTP/GDP were downregulated (Fig. 5A), indicating that inhibiting ME2 has a detrimental effect on energy production and cellular metabolism.

Overall, the results of the mass analysis experiment conducted by silencing ME2 or administering a ME2 inhibitor indicate that ME2 regulates multiple metabolic pathways, including glycolysis, the utilization of glutamine and glutamate, the biosynthesis of essential molecules, and glycogenolysis (Figs. 2 and 5). The findings provide important insights into the role of ME2 in cellular metabolism and may have implications for the development of therapies targeting ME2 in AML, where dysregulation of cellular metabolism is a hallmark.

### Antileukemic efficacy of ME2 inhibitor EA or Na_2_EA against disseminated AML immune-deficient mice

**Fig. 6:**
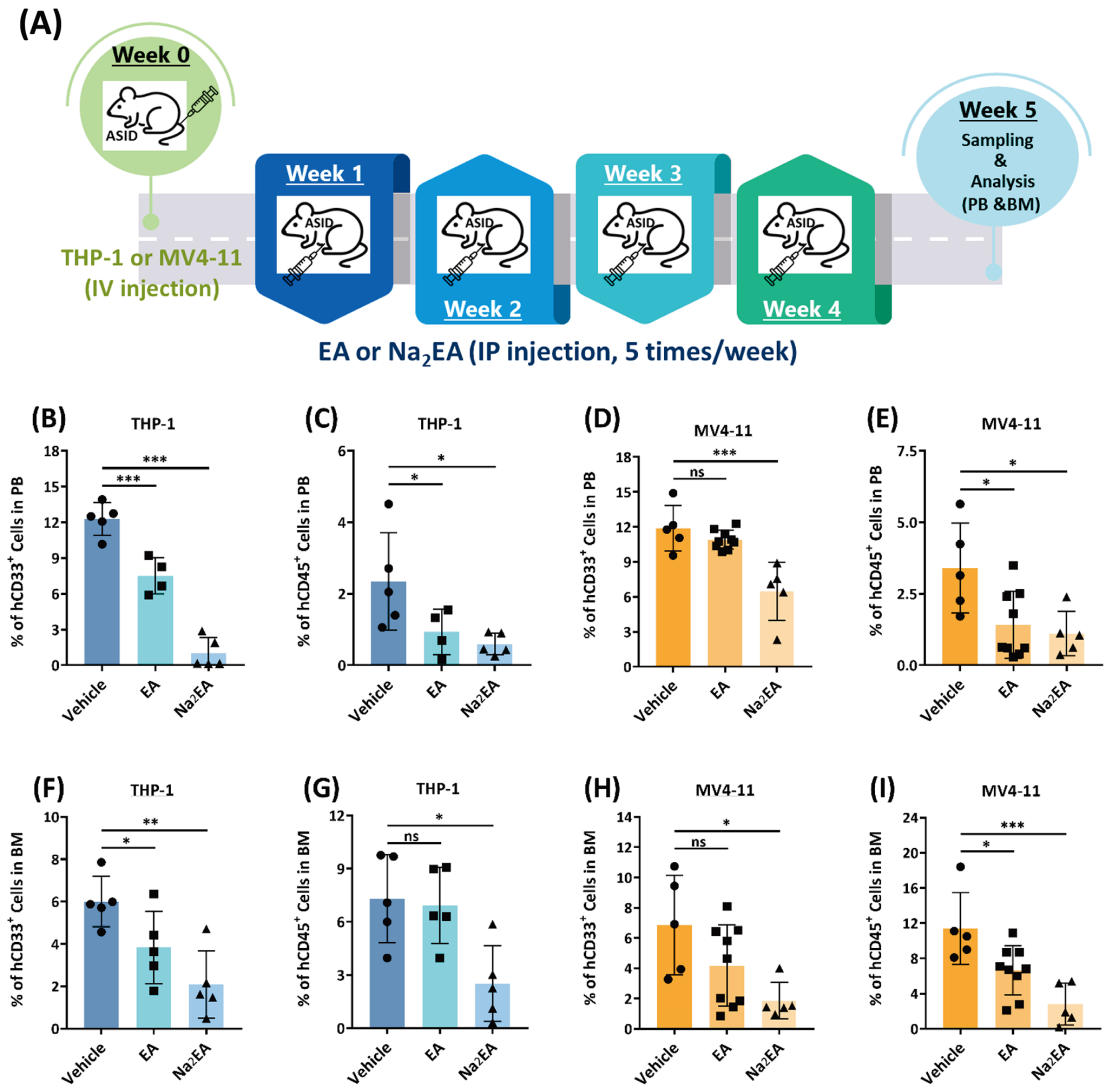
Inhibition of ME2 in THP-1 and MV4-11 cell line-derived xenograft model studies. **(A)** Diagram illustrating the administration of the ME2 inhibitors EA or Na_2_EA to ASID mice implanted with THP-1 and MV4-11 cells. **(B)** and **(C)** Percentage of CD33 + and CD45 + cells, respectively, in EA or Na_2_EA-treated THP-1 cells in the peripheral blood (PB) of mice after five weeks. **(D)** and **(E)** Percentage of CD33 + and CD45 + cells, respectively, in EA or Na_2_EA-treated MV4-11 cells in the peripheral blood (PB) of mice after five weeks. **(F)** and **(G)** Percentage of CD33 + and CD45 + cells, respectively, in EA or Na_2_EA-treated THP-1 cells in the bone marrow (BM) of mice after five weeks. **(H)** and **(I)** Percentage of CD33 + and CD45 + cells, respectively, in EA or Na_2_EA-treated MV4-11 cells in the bone marrow (BM) of mice after five weeks

We evaluated the antileukemic efficacy of ME2 inhibitors EA (non-ionic form) and Na_2_EA (ionic form) in THP-1 and MV4-11 disseminated AML ASID mouse models (Fig. 6A). After three weeks of xenotransplantation, the antileukemic efficacy of EA or Na_2_EA against THP-1 and MV4-11 immune-deficient mice was determined by monitoring PB CD33 + or CD45 + cells (Figure S10). Both EA and Na_2_EA treatment led to a decrease in AML cells, as observed by monitoring CD33 + but not CD45 + cells in the PB of THP-1 mice (Figures S10A and S10B, respectively). EA or Na_2_EA treatment significantly reduced the number of MV4-11 cells in AML ASID mice, whereas the proportion of CD33 + and CD45 + cells was less than 1% (Figures S10C and S10D, respectively).

In addition, the antileukemic efficacy of Na_2_EA was determined in THP-1 and MV4-11 AML mice five weeks after xenotransplantation by monitoring PB and BM CD33 + or CD45 + cells (Figures S11 and S12, respectively). After five weeks of xenotransplantation, the antileukemic efficacy of EA or Na_2_EA against THP-1 ASID mice was significant when monitoring PB CD33 + or CD45 + cells (Fig. 6B C, respectively); however, only Na_2_EA is capable of reducing THP-1 AML cells in the bone marrow when monitoring both BM CD33 + and CD45 + AML cells (Fig. 6D and E, respectively).

EA did not demonstrate significant antileukemic efficacy against MV4-11 ASID mice when monitoring CD33 + or CD45 + cells in peripheral blood (Fig. 6F and G, respectively); whereas only Na_2_EA exhibited antileukemic efficacy against MV4-11 ASID mice when monitoring CD33 + or CD45 + cells in peripheral blood (Fig. 6F and G, respectively) and bone marrow (Fig. 6H and I, respectively). These results suggested that Na_2_EA has a positive effect on inhibiting the development of leukemia, which was attributed to ME2-mediated energy metabolism dysregulation (Figs. 4 and 5).

## Discussion

**Fig. 7:**
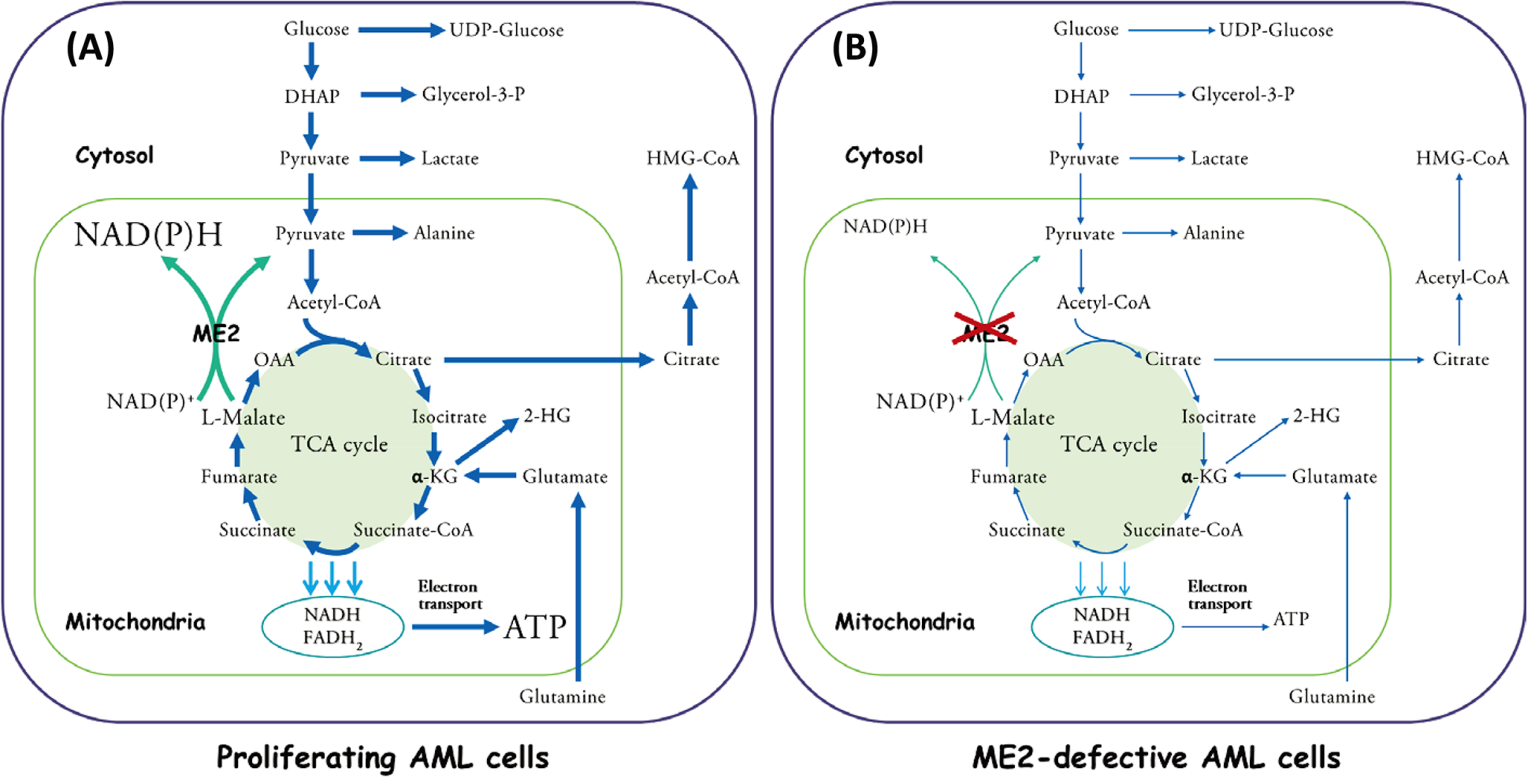
ME2-involved metabolism in AML cells. **(A)** ME2 and its associated metabolic functions in proliferating AML cells **(B)** Metabolism inhibition in ME2-defective AML Cells (ME2 silencing or ME2-inhibitor treatment)

Multiple studies have demonstrated that ME2 plays a crucial role in the development of numerous human cancers, including melanoma and glioma [16, 19, 20, 28, 31, 35–37]. Our current work highlights that ME2, which is involved in energy metabolism and possesses antioxidative activity, is necessary for the survival of AML cells (Fig. 7A). By silencing ME2 or administering ME2 inhibitors (EA or Na_2_EA), inhibition of ME2 reduces ATP and NAD(P)H production, which are essential for cellular energy metabolism and redox balance. The decrease in NADPH production increases reactive oxygen species (ROS), which can induce apoptosis in AML cells (Fig. 7B). Our findings proved conclusively that targeting ME2 disrupted the energy metabolism and redox equilibrium in AML cells, leading to antileukemic effects against AML.

In cancer cells, metabolic adaptation is crucial to meet the high demand for energy and biosynthesis required for rapid cell growth and division. The Warburg effect, also known as aerobic glycolysis, is a metabolic adaptation that provides energy for cancer cells even in the presence of oxygen [10, 38]. Additionally, cancer cells utilize glutamine as an alternative source of energy and precursors for biosynthesis [16, 19, 25, 31, 32]. Glutamine is the most abundant amino acid in the bloodstream, and a high consumption of glutamine has been observed in a variety of cancers. Consequently, plasma glutamine levels were found to be significantly lower in cancerous tissue than in healthy tissue [25]. ME2 is an enzyme involved in the conversion of L-malate to pyruvate and NADH/NADPH in the mitochondria, which is necessary for continuous ATP generation and NADPH production to combat reactive oxygen species (ROS). A mass analysis with [U-^13^ C]glutamine for the study of ME2 silencing has revealed ME2’s involvement in this pathway of glutamine metabolism via the TCA cycle [32]. Therefore, we account that ME2 activity may drive glutamine and glutamate metabolism to satisfy the energy demands of growing AML cells (Fig. 7A). In our study, we discovered that inhibition of ME2 increases glutamine and glutamate levels while decreasing the levels of TCA cycle intermediates, indicating that glutamine metabolism is slowed, which may result in a reduction of the TCA cycle intermediate anaplerosis (Fig. 7B). Consequently, ME2 may also play a significant role in the anaplerosis of glutamine and glutamate-derived TCA cycle intermediates.

Recent research reveals the role of ME2 in regulating the production of 2-HG, a metabolite recognized as an oncometabolite, showing that ME2 upregulates the production of 2-HG via a catalytic mechanism that requires pyruvate and NADPH and is distinct from the mutant isocitrate dehydrogenase-catalyzed reaction [37]. This coincides with our observation that when ME2 is silenced, the 2-HG levels are reduced (Fig. 2C). Additionally, the silence of ME2 also cause a reduction in the levels of alanine and lactate, possibly due to a reduction in cellular pyruvate (Fig. 7B). The precursor for cholesterol biosynthesis, HMG-CoA, may also fall as a result of a drop in citrate levels (Fig. 7B). The silence of ME2 may also alter glycolysis, as indicated by the decreased levels of glycolytic intermediates (Fig. 2D). When DHAP level was dropped, the skeleton of triacylglycerol, glycerol-3-phosphate, which is derived from DHAP, was diminished (Fig. 7B). Glycogen synthesis may also be altered, as indicated by the reduced UDP-glucose intermediate (Fig. 7B). Overall, our research suggests that ME2 plays an important role in regulating various metabolic pathways and its silence may have significant effects on cellular metabolism.

Xenograft studies have shown that the salt form of embonate, Na_2_EA, is more effective against leukemia than the acidic form, EA, possibly due to differences in bioavailability. However, using a ME2 inhibitor alone may not be sufficient to cure AML, as cancer cells can adapt to altered metabolism. Targeting ME2-involved metabolism, which includes inhibiting ATP generation and increasing ROS production, is still a promising strategy for AML therapy. A proto-oncogene FMS-like tyrosine kinase 3 (FLT3) is involved in the pathogenesis of AML, and the most prevalent FLT3 mutation is an internal tandem duplication (ITD), which enhances ligand-independent auto-phosphorylation and constitutive activation of the receptor [39]. Approximately 25% of all AML cases contain FLT3 gene mutations with ITD [39]. The FLT3-ITD mutation results in a substantial leukemic burden in AML patients, and it is strongly related with a poor prognosis for AML [40]. It is also believed that AML cells are highly dependent on glutamine for their survival [29], and targeting glutamine metabolism has been considered a promising target for the development of anticancer drugs [41]. Combining the glutaminase inhibitor CB-839 with a potent FLT3 inhibitor AC220 has enhanced survival in a patient-derived xenograft mice model of AML [42]. Future research could focus on combining ME2 inhibitors with other chemotherapeutic agents, such as cytarabine and daunorubicin or idarubicin, or with FLT3 or glutaminase inhibitors, to improve the overall efficacy of AML therapy and delay the recurrence of the disease due to chemoresistance.

## Materials & methods

### Cell culture

Human acute myeloid leukemia (AML) MV4-11 (RRID: CVCL_0064) OCI-AML-2 (RRID: CVCL_1619) cell was purchased from American Type Culture Collection (ATCC, Manassas, VA, USA); the human acute myeloid leukemia THP-1 (RRID: CVCL_0006) and HL-60 (RRID: CVCL_0002) cells were purchased from Bioresource Collection and Research Center (BCRC, Hsinchu, Taiwan), and the human embryonic kidney HEK293FT (RRID: CVCL_6911) cell was purchased from Thermo Fisher Scientific (Waltham, MA, USA). MV4-11, THP-1, HL-60, and ODC-AML-2 cells were grown in RPMI-1640 Medium (HyClone™, Cytiva, Marlborough, MA, USA) supplemented with 10% fetal bovine serum (Sigma-Aldrich, St. Louis, MO, USA) and 1% penicillin/streptomycin. HEK293FT cells were grown in Dulbecco’s Modified Eagle Medium (DMEM) (HyClone™, Cytiva, Marlborough, MA, USA) supplemented with 10% fetal bovine serum and 1% penicillin/streptomycin. These cell lines were cultured at 37 °C in a humidified incubator containing 5% CO_2_. All experiments with cell lines were performed in mycoplasma-free cells.

### ME2 silencing and lentiviral infection

Transient transfection of HEK293FT cells with TransIT-VirusGEN® Transfection Reagent (Mirus bio LLC, Madison, WI, USA) generates lentiviral particles. Briefly, HEK293FT cells seeded in a 6-well plate were transfected with 6 µl of transfection reagent (Thermo Fisher Scientific, Waltham, MA, USA) containing 1.35 µg pCMV, 0.15 µg pMD and 1.5 µg of the ME2-targeting shRNA vector. After 48 hours, 2 ml of the virus-containing supernatants were collected. MV4-11, THP-1, and HL-60 cells (5 × 10^5^ cells/ml) were infected with lentiviral particles expressing ME2-specific shRNA for 24 hours in the presence of 8 µg/ml polybrene, followed by puromycin selection for 48 hours. shME2 (TRCN0000294006 and TRCN0000294007) and shCtrl (ASN0000000001) were obtained from the National RNAi Core Facility (Academia Sinica, Taipei, Taiwan). Using RT-qPCR or flow cytometry, the knockdown efficiency was determined. These sequences were: non-targeting control shCtrl: 5’- CCGGAGTTCAGTTACGATATCATGTCTCGAGACATTCGCGAGTAACTGAACTTTTTT-3’ (ASN0000000001); human shME2-1: 5’-ACTGAAGCCTTCAACTATAAT-3’ (TRCN0000294006); human shME2-2: 5’-AGTTCTTACAGAGCTACTAA-3’ (TRCN0000294007).

### Measurement of cellular ATP levels

The ATP content of cells was determined using a CellTiter-Glo® 2.0 Cell Viability Assay (#Cat. G9242; Promega, Madison, WI, USA). On 6 cm plates, the cells (5 × 10^5^/ml) were re-suspended in 100 µL PBS and reacted with an equal volume of the assay buffer. The luminescence was detected using the Biotek® multi-mode microplate reader (Agilent, Santa Clara, CA, USA). The ATP content was also detected using a BioTracker™ ATP-Red Live cell dye (#SCT-045, Sigma-Aldrich, St. Louis, MO, USA) was used to measure ATP levels in the cell. The cells (5 × 10^5^/ml) were washed in 200 µL phosphate-buffered saline (PBS) buffer and centrifuged for 5 min at 300xg. The cells were resuspended in 99.9 µL PBS buffer with 0.1 µL ATP probe (10 mM), and a 37 °C humidified incubator was used to incubate the cells for 30 min. Finally, the Cells were washed with PBS buffer and subjected to fluorescence-activated cell sorter (FACS) analysis. Using a BD Accuri C6 Plus flow cytometer, data was collected (BD Biosciences, San Jose, CA, USA).

### Live cell fluorescence microscopy

ATP was measured using a BioTracker™ ATP-Red Live cell dye (#SCT-045, Sigma-Aldrich, St. Louis, MO, USA), and nucleic acid was detected using bisBenzimide H 33,342 trihydrochloride dye (Sigma-Aldrich, St. Louis, MO, USA). At 37 °C, cells (1 × 10^6^/500 µL) were stained with 5 µM ATP-Red dye for 1 h and 2 µg/ml H 33,342 for 20 min. After centrifuging cells at 800 rpm for 3 min, discard the supernatant and resuspend the pellet in 20 µl PBS buffer. Transfer the cell suspension to a microscope slide. The fluorescent and color bright field cell images were acquired and analyzed using a Lionheart FX automated microscope (BioTek®, Agilent, Santa Clara, CA, USA).

### Apoptosis

A 24-well plate containing RPMI medium was seeded with cells with or without Na_2_EA treatment, or stable cell lines containing shCtrl or shME2. After 24 h, the medium was replaced. After 48 h of incubation, the Annexin V Apoptosis Detection Kits (#cat. 88-8005-72, eBioscience, Thermo Fisher Scientific, Waltham, MA, USA) were used to assess apoptosis by flow cytometry. The cells (5 × 10^5^/ml) were washed in 200 µL of binding buffer and centrifuged for 5 min at 300xg. The cells were resuspended in a binding buffer (195 µL) with 5 µL of Annexin V-FITC after discarding the supernatant. After 10 min of room temperature incubation, the cells were centrifuged at 300xg for 5 min. The cells were resuspended in a binding buffer (195 µL) with 5 µL of propidium iodide after discarding the supernatant. The FACS analysis of 10,000 events was performed on a BD Accuri C6 Plus system, (BD Biosciences, San Jose, CA, USA).

### ROS measurement

ROS content was measured using an intracellular ROS kit (#cat.MAK143, Sigma-Aldrich, St. Louis, MO, USA) according to the manufacturer’s protocol. Cells were cultured at a density of 5 × 10^4^ cells per well in 96-well black plates. Briefly, 100 µL of Master Reaction Mix was added to each well, followed by an hour of incubation at 37^o^C in an incubator. The FACS analysis of 10,000 events was performed on a BD Accuri C6 Plus system, (BD Biosciences, San Jose, CA, USA).

### Measurement of cellular NADPH levels

NADP/NADPH-Glo™ Assay kit (#Cat. G9081, Promega, Madison, WI, USA) was used to evaluate the NADPH assay. On 6-cm plates, cells were incubated RPMI with 150 µM EA for 48 h at 37 °C and 5% CO_2_. 5 × 10^5^/ml of cells were washed with PBS buffer, resuspended in 50 µl of PBS buffer, and lysed with 50 µl of base solution. Mix the plate briefly to guarantee homogeneity and cell lysis. 50 µl of each sample was transferred to an empty well and incubated at 60 °C for 15 min. The plate was equilibrated at room temperature for 10 min before 50 µl of HCl/Trizma® solution was added. The samples were treated with 100 µl of detection reagent, followed by a 60-minute incubation at room temperature. Using a multimode microplate reader (BioTek®, Agilent, Santa Clara, CA, USA), the level of NADPH was determined.

### Measurement of cellular NAD^+^ and NADH levels

The levels of NAD^+^ and NADH were measured using the NAD/NADH-Glo™ Assay (#Cat. G9071; Promega, Madison, WI, USA). On 6-cm plates, cells were incubated RPMI with 150 µM EA for 48 h at 37 °C and 5% CO_2_. The cells (5 × 10^5^/ml) were washed and suspended in 100 µl of PBS buffer before 50 µl of base solution was added to lyse the cells. For NAD^+^ measurement, 50 µl of each sample was transferred to an empty well, followed by the addition of 25 µl of 0.4 N HCl and 15 min of incubation at 60 °C. The plate was equilibrated at room temperature for 10 min before 25 µl of Trizma® solution was added. For NADH measurement, 50 µl of each sample was transferred to an empty well and incubated at 60 °C for 15 min. The plate was equilibrated at room temperature for 10 min before 50 µl of HCl/Trizma® solution was added. The samples were treated with 100 µl of detection reagent, followed by a 60-minute incubation at room temperature. Using a multimode microplate reader (BioTek®, Agilent, Santa Clara, CA, USA), the level of NAD^+^ and NADH was determined.

### Measurement of cellular pyruvate levels

Pyruvate Colorimetric/Fluorometric Assay Kit was used to measure the cellular pyruvate levels (K609-100, BioVision, Milpitas, CA, USA). On 6-cm plates, MV4-11, THP-1, and HL-60 cells were cultured for 48 h at 37 °C, 5% CO_2_ with RPMI containing 150 µM EA. With 100 µL of PBS buffer, the cells were harvested and sonicated. After 13,000 rpm of centrifugation, the supernatant was deproteinized with a 10 kDa spin column (Nanosep, Acrodisc® syringe filter, Pall Life Sciences, New York, United States) and reacted with a reaction mixture. Fluorescence data (Ex/Em at 535/587 nm) was gathered using a multimode microplate reader (BioTek®, Agilent, Santa Clara, CA, USA).

### Measurement of cellular glutamate levels

Utilizing the Glutamine/Glutamate-Glo™ Assay kit (#Cat. J8021; Promega, Madison, WI, USA), the levels of glutamate were measured. On 6-cm dishes, cells were treated with 150 µM EA at 37 °C, 5% CO_2_ and 5% humidity. After 48 h of treatment, cells were washed twice with 200 µL PBS, resuspended in 30 µL PBS, and then combined with 15 µL Inactivation Solution I. (0.3 N HCl). After 5 min of shaking, 15 µL of Tris Solution I (450 mM Tris-HCl, pH 8.0) was added to each sample and mixed for 1 min. Using the 10 kDa spin column (Nanosep, Acrodisc® syringe filter, Pall Life Sciences, NY, USA), the samples were deproteinized, and 50 µL of each sample was transferred into a white 96-well plate. In each well, 50 µL of Glutamate Detection Reagent was added and mixed with the sample by shaking the plate for 1 min. After incubating for 60 min at room temperature, the levels of glutamate were measured using the BioTek® multimode microplate reader to detect luminescent signals (Agilent, Santa Clara, CA, USA).

### Oxygen consumption rate measurements

Using Seahorse XF Extracellular Flux Analyzer XF24, the oxygen consumption rate (OCR) of cells was monitored in real-time (Agilent, Santa Clara, CA, USA). All tests were conducted according to the manufacturer’s guidelines. Briefly, 50,000 cells per well were coated with Cell-Tak (#Cat. 354,240, Corning, NY, USA) for 30 min on Seahorse XF24 Cell Culture Miniplate (Seahorse Biosciences, Agilent, Santa Clara, CA, USA). Prior to the assay, cells were equilibrated in a non-CO_2_ incubator for 30 min with basal medium (Seahorse Biosciences, Agilent, Santa Clara, CA, USA) containing 1 mM pyruvate, 4 mM glutamine, and 1 mg/mL D-glucose. To establish a stable baseline OCR, the OCR under baseline measurements was measured at the beginning of the assay, prior to the injection of compounds. Following injection of stressed conditions (ATP-linked respiration by 1 µM oligomycin, spare respiratory capacity by 3 µM FCCP, proton leak by 0.5 µM rotenone, and 0.5 µM antimycin A), mitochondrial efficiency was evaluated. Using a Seahorse XFe24 Analyzer and the Seahorse XF Cell Mito Stress Test, the oxygen consumption rate (OCR) was determined (Agilent, Santa Clara, CA, USA). The experimental data were analyzed using the Wave2.6 control program (Agilent, Santa Clara, CA, USA).

### Cell viability assay

In a 12-well plate, cells (5 × 10^5^ cells/ml) were seeded and treated with Na_2_EA for 48 h. Using the CellTiter-Fluor™ Cell Viability Assay, cell viability was determined (#Cat. G6080, Promega, Madison, WI, USA). Using a BioTek® multi-mode microplate reader with Ex/Em = 490/505 nm, the viability of the cells was measured (Agilent, Santa Clara, CA, USA). To assess the viability of ME2-knockdown cell lines, 5 × 10^5^ cells/ml were plated in a 6-centimeter dish. After 24, 48, and 72 h, the viability of the cells was examined. Using a FACSCOPE B cell counter (Curiosis, Korea) with a 4-channel disposable slide, the cell counts was observed. Each channel contained 20 µl of cell suspensions blended with an equal volume of trypan blue (Sigma-Aldrich, St. Louis, MO, USA).

### Western blot analysis

Cells were lysed and homogenized in RIPA lysis buffer at 4 °C, and proteins were separated by sodium dodecyl sulfate-polyacrylamide gel electrophoresis (SDS-PAGE). After transferring the proteins to a polyvinylidene difluoride (PVDF) membrane (Millipore, USA), the antibodies against β-actin (Arigo Biolaboratories, Taiwan) and human ME2 (MDbio Inc., Taiwan) was used to detect the amount of proteins utilizing an ImageQuant™ LAS 4000 mini imager (GE Healthcare Life Sciences, USA).

### Measurement of cellular ME2 protein levels using flow cytometry

AML cells were washed in staining buffer (#Cat. 00-4222-26, eBiosciences) and 0.1% BSA prior to a 45-minute incubation with a non-conjugated human-ME2-specific antibody (MDbio Inc., Taiwan). The cells were then washed twice with 0.1% BSA-containing staining buffer. For five minutes, centrifuge the cells at 300xg. Throw away the supernatant and resuspend the pellet in 200 µL staining buffer containing 0.1% BSA. Alexa Fluor 488 anti-rabbit antibody (Sigma-Aldrich, St. Louis, MO, USA) was added as a secondary antibody and incubated at room temperature for 15 min. The cells were then washed twice with 0.1% BSA-containing staining buffer. For five minutes, centrifuge the cells at 300xg. Throw away the supernatant and resuspend the pellet in 200 µL staining buffer containing 0.1% BSA. FITC isotype was used as the control (#Cat. 555,748, BD Biosciences, San Diego, CA, USA). Using a BD Accuri C6 Plus flow cytometer, data were collected and analyzed with BD Accuri C6 Plus Software (BD Biosciences, San Jose, CA, USA).

### Mass spectrometry

MV4-11 or HL-60 cells (1 × 10^7^ cells) were added to 1 mL 80% methanol to extract cellular metabolites and precipitate proteins. Samples were centrifuged with 12,000xg for 10 min at 4 °C. Nitrogen gas was used to evaporate supernatant, which was then analyzed with Waters ultra-high-performance liquid chromatography coupled with Waters Xevo TQ-S MS (Waters Corp. Milford, MA, USA). Mass was operated in negative and positive with multiple reaction monitoring modes. Using a tuning method, the major MS/MS fragment patterns of each analysis were determined. The optimized parameters were as follows: capillary voltage at 1 kV, desolvation temperature at 500 °C, source temperature at 150 °C, and gas flow at 1000 L/h. The chromatographic separation was achieved on a BEH C18 column (100 × 2.1 mm, the particle size of 1.7 μm; Waters Corp.) at 45 °C with solution A (water with 10 mM tributylamine and 15 mM acetic acid) and solution B (50% acetonitrile with 10 mM tributylamine and 15 mM acetic acid), and the flow rate was set at 0.4 mL/min.

### Xenograft AML mice model

All animal research was approved by the Institutional Animal Care and Use Committee of the National Laboratory Animal Center (NLAC) and conducted in accordance with the Association for Assessment and Accreditation of Laboratory Animal Care (AAALAC) guidelines for mice. In the laboratory at NLAC, all animal experiments were conducted. Eight-week-old male NOD.Cg-*Prkdc*^*scid*^*Il2rg*^*tm1Wjl*^/YckNarl mice (Advanced Severe Immunodeficiency mice, ASID) were separated into distinct groups for each type of cell line, and at least five mice were utilized in each group. Mice were xenografted by intravenous injection of 5 × 10^6^ MV4-11 or THP-1 cells that contained shCtrl, shME2-1, or shME2-2. In order to analyze human leukemia cell engraftment, peripheral blood (PB) was extracted from the facial vein of mice after 21 days, and bone marrow (BM) was extracted from mice after 35 days.

To identify human leukemia cells, BM and PB cells extracted from xenograft mice were stained with particular markers (CD33 and CD45). For CD33 + and CD45 + staining of hematopoietic cell populations, the BM and PB samples were filtered through a 40-mm mesh to obtain single cell suspensions and depleted of red blood cells using RBC lysis buffer (#Cat. 00-4333-57, eBioscience) for 15 min at room temperature. Cells (1 × 10^6^ cells/sample) were incubated for 1 h at room temperature with the appropriate dilution (1 µg/l) of fluorescent antibody conjugates. CD45-FITC (#Cat. 11-0459-42, eBioscience), CD33-PE (#Cat. 555,450, BD Bioscience), FITC isotype control (#Cat. 555,748, BD Bioscience), and PE isotype control (#Cat. 555,749, BD Bioscience) antibody conjugates were utilized. Using human CD33-PE and CD45-FITC, the number of human leukemic cells (hCD33+/hCD45 + cells) was quantified utilizing BD Accuri C6 Plus flow cytometry (BD Biosciences, San Jose, CA, USA).

For the in vivo EA or Na_2_EA animal experiments, leukemic cell-xenografted mice were divided into four groups (MV4-11 with vehicle, MV4-11 with EA or Na_2_EA, THP-1 with vehicle, and THP-1 with EA or Na_2_EA) and at least five mice were used for each group. Intraperitoneal (IP) injections of vehicle or EA or Na_2_EA (4 mg/kg) were administered to xenografted mice five days per week for four weeks. The peripheral blood (PB) of mice was extracted from the facial vein 21 days after cell transplantation. The bone marrow of mice was collected 35 days after cell transplantation. The quantity of human leukemic cells (hCD33+/hCD45 + cells) was then determined by BD Accuri C6 Plus flow cytometry (BD Biosciences, San Jose, CA, USA).

### CD45^+^ AML cell isolation

Cryopreserved mononuclear cell fractions of AML patients were thawed, resuspended in FBS (2%), supplemented with DNase I (20 Units/mL), and incubated on 37 °C for 15 min. Using CD45 + antibody-labeled magnetic beads (#Cat. 480,030, BioLegend), CD45 + AML cells were subsequently isolated. The cells were counted, the concentration was adjusted to 1 × 10^8^ cells/ml, and the cells were resuspended in MojoSort™ buffer (#Cat. 480,017, BioLegend) by adding up to 4 ml in a 5 ml polypropylene tube. Transfer 100 µL of cell suspension (1 × 10^7^ cells) to a new tube, add 5 µl Human TruStain FcX™ (#Cat. 422,301, BioLegend), and incubate for 10 min on ice. Wash the cells by adding up to 4 ml of MojoSort™ buffer and centrifuging them at 300xg for 5 min. Throw away the supernatant and resuspend the pellet in 4 ml MojoSort™ Buffer. Add the CD45 antibody-labeled beads (10 µL), mix thoroughly, and incubate for 15 min on ice. Add up to 4 ml of MojoSort™ buffer to the cells and centrifuge them at 300xg for 5 min. Throw away the supernatant and resuspend the pellet in 2.5 ml MojoSort™ Buffer. Place the tube in the magnet for 5 min and repeat twice on the fraction labeled with beads; then the cells are ready for analysis.

### Statistical analysis

The data presented denote means ± standard deviations (mean ± SD). GraphPad Prism was used to calculate and plot the means and standard deviations (version 7; GraphPad Software, USA). Statistical analysis was performed by Student’s *t*-test at significance levels of *p < 0.05, **p < 0.01, and ***p < 0.001.

### Electronic supplementary material

Below is the link to the electronic supplementary material.

Supplementary Material 1

## Data Availability

Not applicable.
