# Supplementary material for: Targeting human mitochondrial NAD(P)+-dependent malic enzyme (ME2) impairs energy metabolism and redox state and exhibits antileukemic activity in acute myeloid leukemia
Source: Cell Oncol (Dordr). 2023 Apr 20;46(5):1301–16. doi: 10.1007/s13402-023-00812-x (PMC10618384; doi:10.1007/s13402-023-00812-x)
Supplement: Supplementary file 1 — Supplementary Material 1 [file 13402_2023_812_MOESM1_ESM.pdf]

Supplementary Information for

Targeting human mitochondrial NAD(P)$^{+}$-dependent malic enzyme (ME2) impairs energy metabolism and redox state and exhibits antileukemic activity in acute myeloid leukemia

Kun-Chi Chen, I-Hsin Hsiao, Yu-Nan Huang, Yu-Tung Chou, Yung-Lung Chang, Yi-Chun Lin, Ju-Yi Hsieh, Guang-Yaw Liu, and Hui-Chih Hung*

Correspondence: Hui-Chih Hung

E-mail: hchung@dragon.nchu.edu.tw

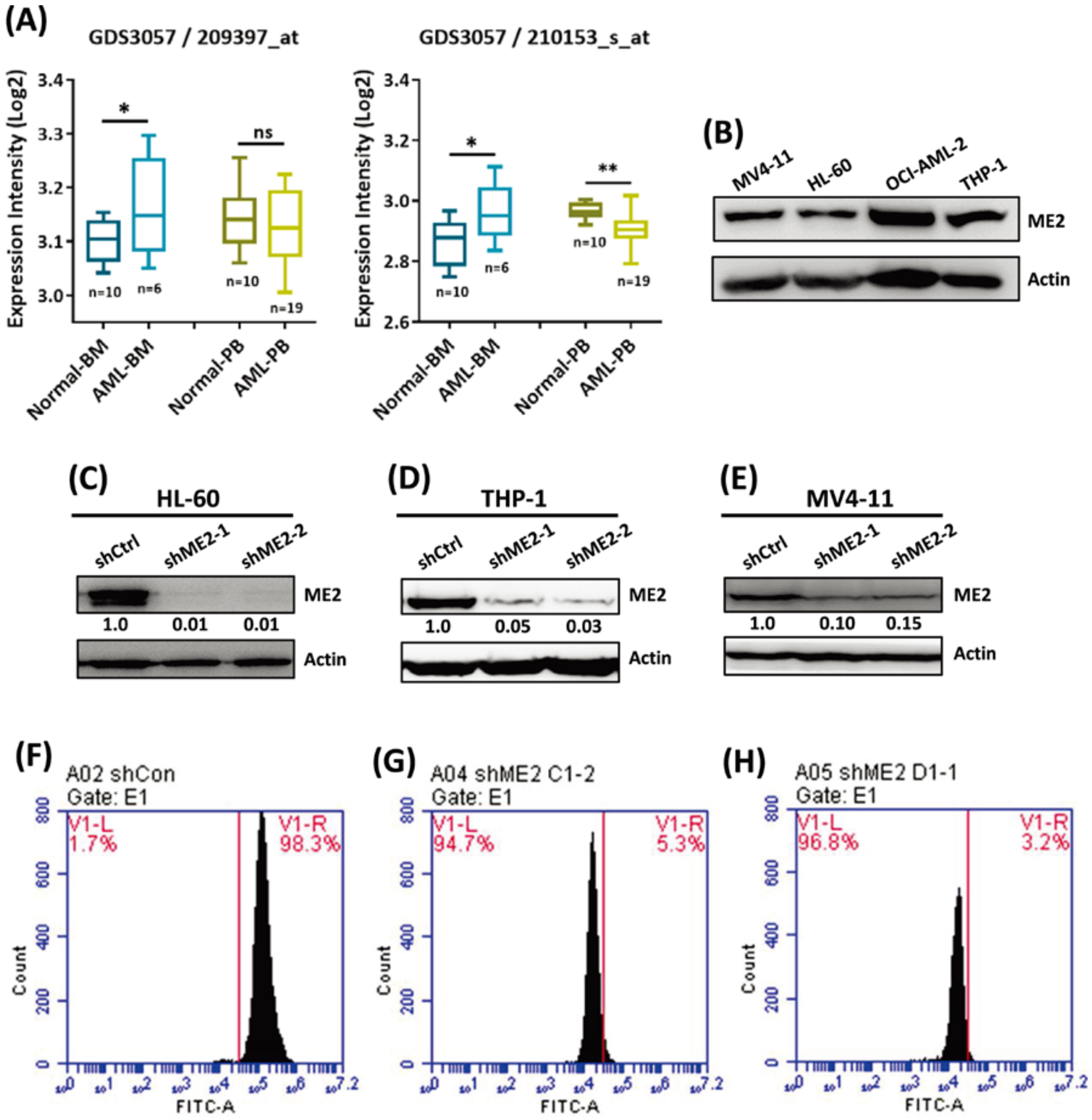

Figure S1. ME2 expression and silence in AML samples or cells. (A) ME2 expression levels in the microarray datasets GDS3057/209397_at and GDS3057/210153_at, which were retrieved from the Gene Expression Omnibus (GEO). Two groups of data (normal and AML) were analyzed from the bone marrow (BM) or peripheral blood (PB). The bar graphs depict the expression intensity of ME2 mRNA (mean ± SD). $^{*}p < 0.05$, $^{**}p < 0.01$, and $^{***}p < 0.001$. p values were calculated using the Student's t-test. **(B)** ME2-expressing AML cell lines. **(C)**, **(D)**, and **(E)** Immunoblotting revealed the residual amounts of ME2 in ME2-silenced HL-60, THP-1, and MV4-11 cells, respectively. **(F)**, **(G)**, and **(H)** Using flow cytometry to determine the residual ME2 levels in ME2-silenced HL-60, THP-1, and MV4-11 cells, respectively.

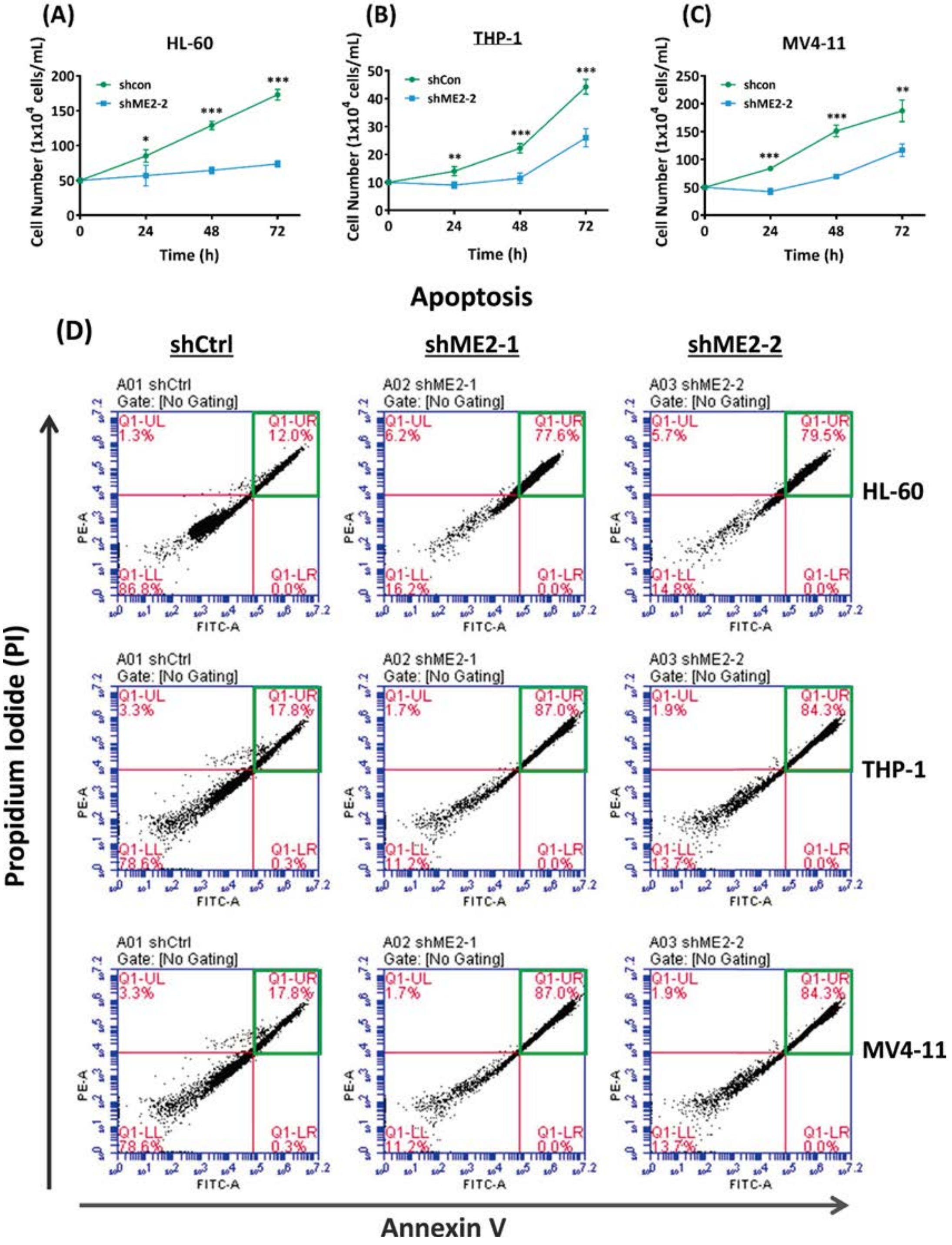

Figure S2. Cell proliferation and apoptosis of ME2-silenced AML cells. (A), **(B)**, and **(C)** Growth curves of ME2-silenced HL-60, THP-1, and MV4-11 cells, respectively. **(D)** Representative graphs demonstrated the flow cytometric analysis of shME2-induced cellular apoptosis.

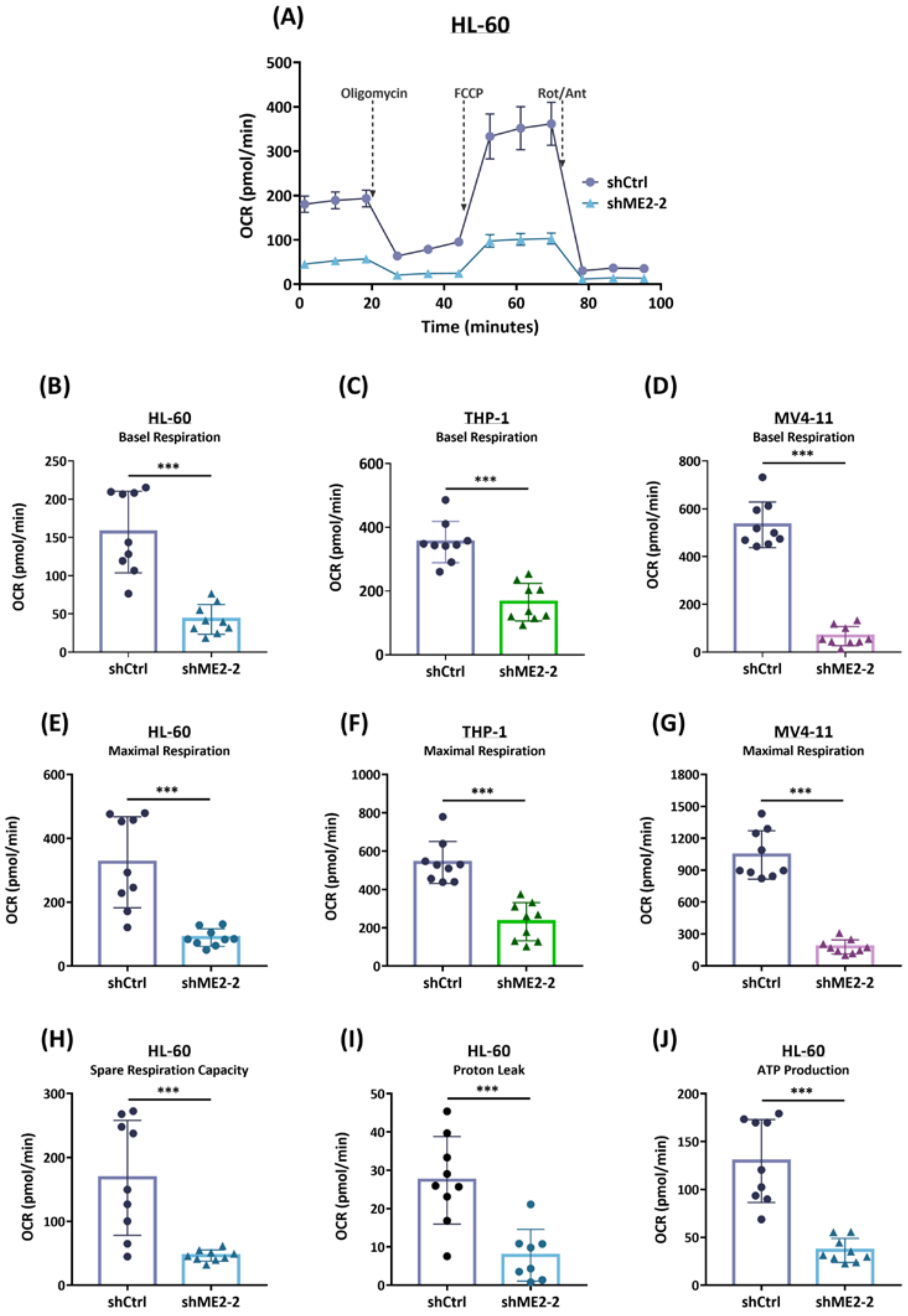

Figure S3. Oxygen consumption rate (OCR) measurement of ME2-silenced AML cells. (A) The OCR in ME2-control (shCtrl) and ME2-knockdown (shME2) HL-60 cells. **(B)**, **(C)** and **(D)** The basal respiration of ME2-silenced HL-60, THP-1, and MV4-11 cells, respectively. **(E)**, **(F)** and **(G)** The maximal respiration of ME2-silenced HL-60, THP-1, and MV4-11 cells, respectively. **(H)** The spare respiration capacity of shME2-HL-60 cells. **(I)** The proton leak of shME2-HL-60 cells. **(J)** ATP production in shME2-HL-60 cells. The bar graphs illustrate the basal and maximal respiration rates, spare respiration capacity, proton leak, and ATP production (multiple numbers in each group, mean ± SD). *p < 0.05, **p < 0.01, and ***p< 0.001. p values were calculated using the Student's t-test.

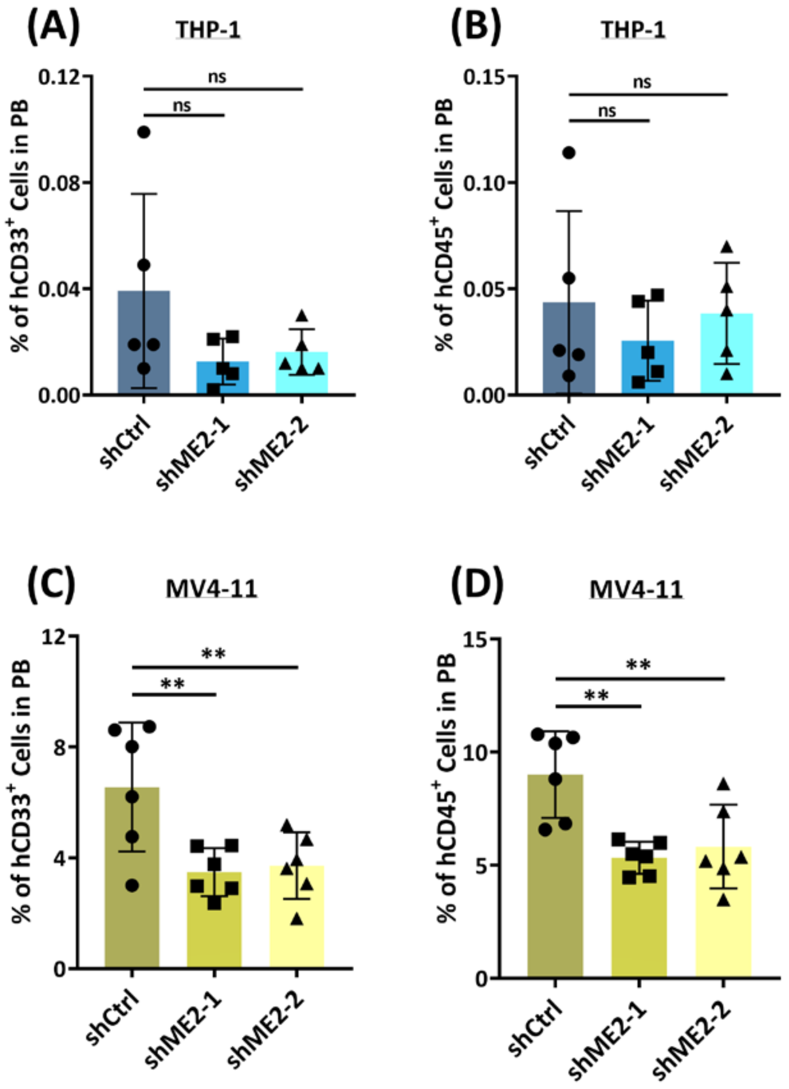

Figure S4. Analysis of the engraftment efficiency of ME2-silenced THP-1 and MV4-11 AML cells after three weeks of xenotransplantation. **(A)** and **(B)** Percentage of CD33+ and CD45+ cells, respectively, in shME2-THP-1 cells in the peripheral blood (PB) of mice after three weeks. **(C)** and **(D)** Percentage of CD33+ and CD45+ cells, respectively, in shME2-MV4-11 cells in the peripheral blood (PB) of mice after three weeks.

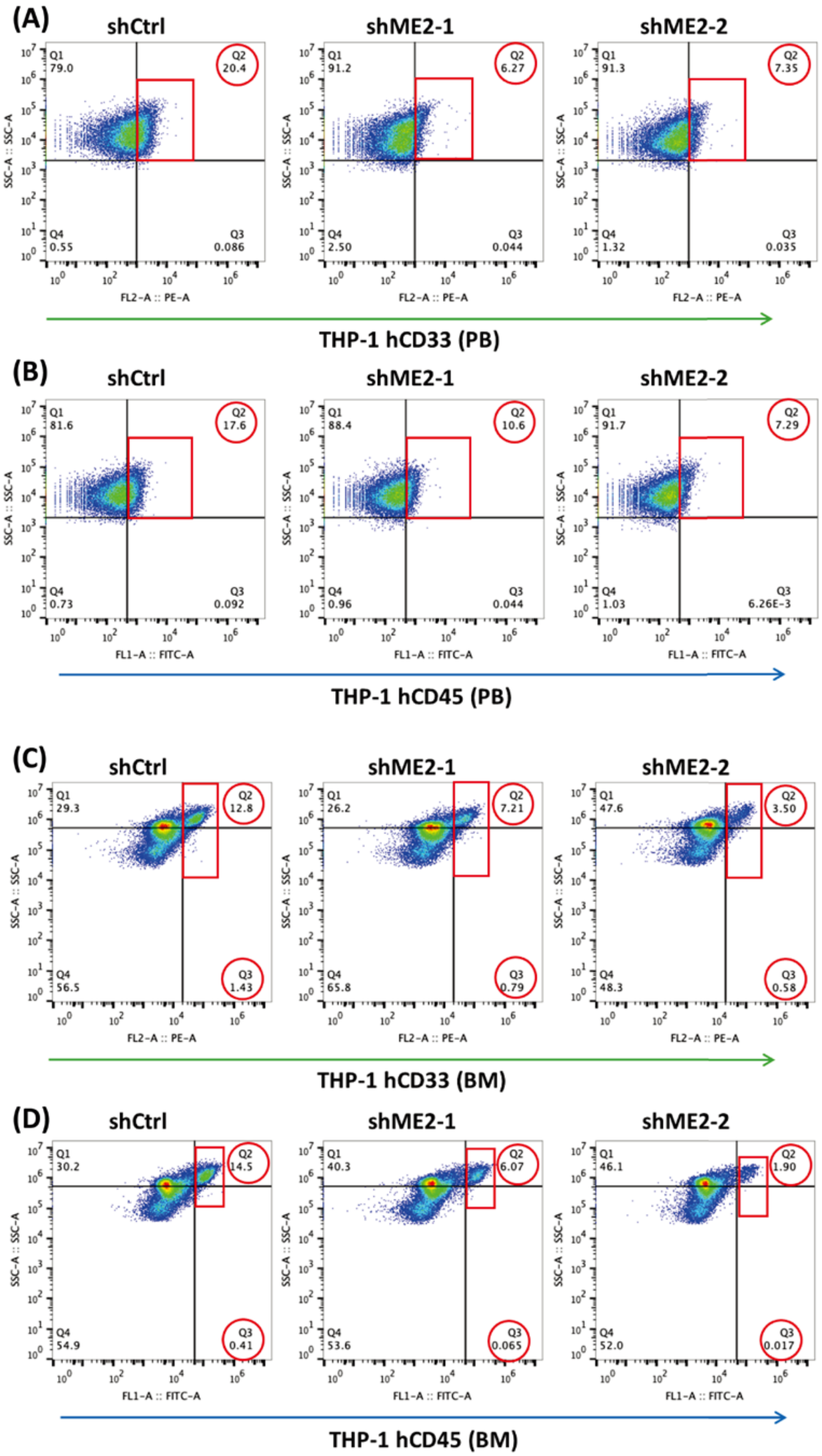

Figure S5. Representative graphs for the flow cytometric analysis of human CD33+ or CD45+ cells from ASID mice with ME2-silenced THP-1 cells. (A) and **(B)** Flow cytometric analysis of the CD33+ and

CD45+ cells, respectively, in the peripheral blood (PB) shME2-THP-1 cells of mice. **(C)** and **(D)** Flow cytometric analysis of the CD33+ and CD45+ cells, respectively, in the bone marrow (BM) shME2-THP-1 cells of mice.

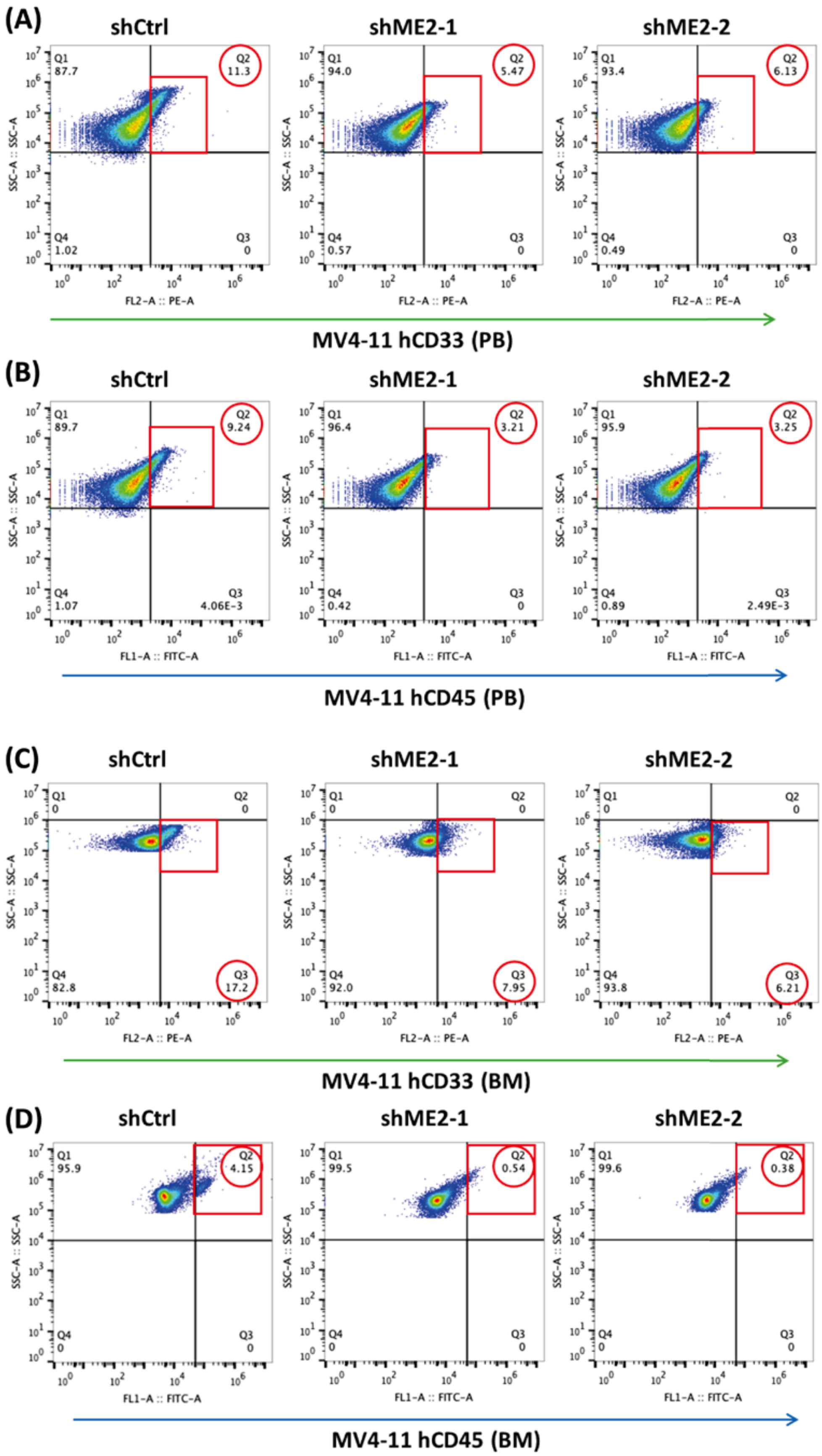

Figure S6. Representative graphs for the flow cytometric analysis of human CD33+ or CD45+ cells from ASID mice with ME2-silenced MV4-11 cells. (A) and **(B)** Flow cytometric analysis of the CD33+ and

CD45+ cells, respectively, in shME2-MV4-11 cells the peripheral blood (PB) of mice. **(C)** and **(D)** Flow cytometric analysis of the CD33+ and CD45+ cells, respectively, in shME2-MV4-11 cells in the bone marrow (BM) of mice.

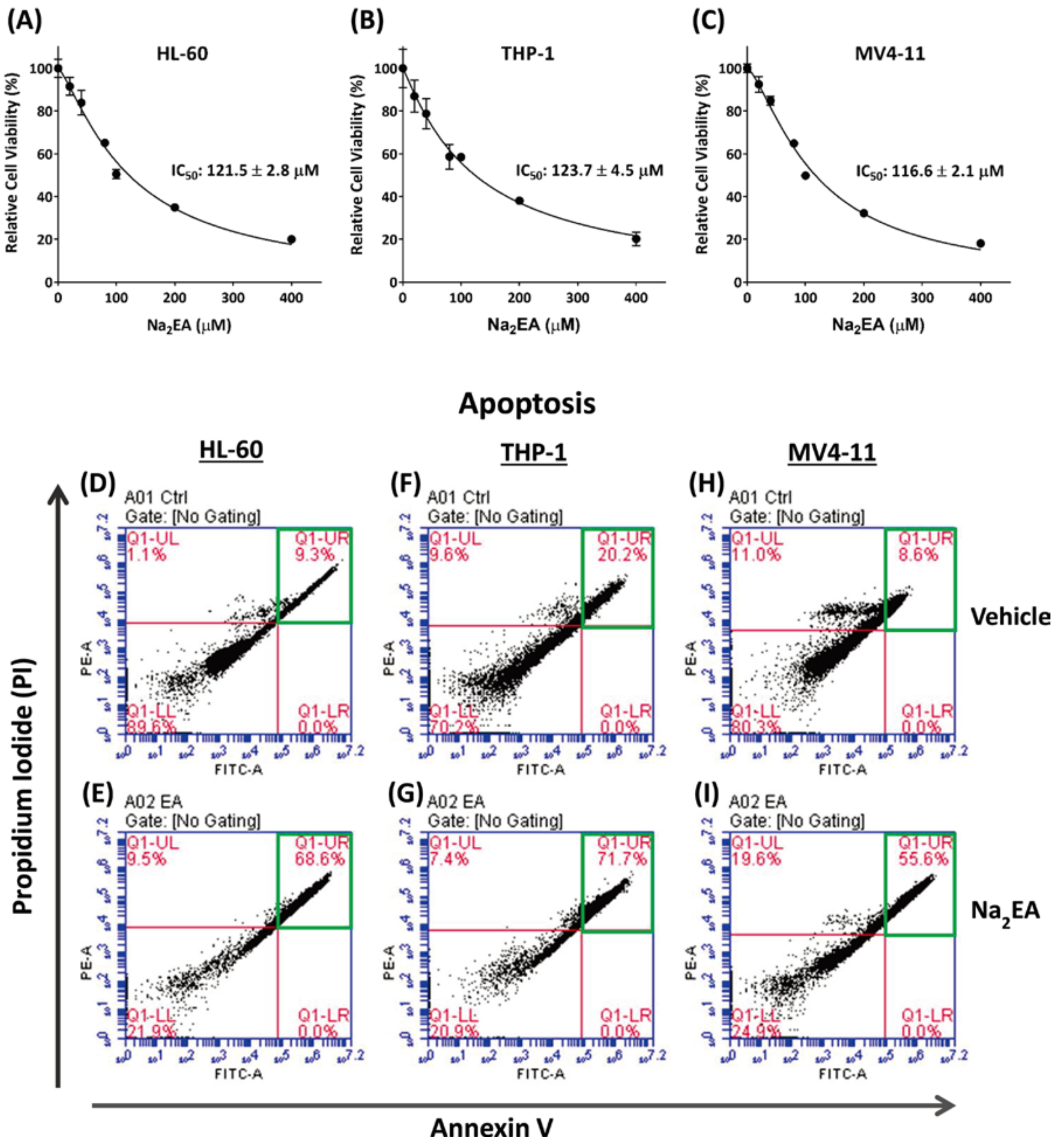

Figure S7. Cell viability after treatment with Na_2EA.

Cell viability was determined at various concentrations of Na_2EA and the IC_{50} value was indicated in the figure (n=3 in each group, mean ± SD). **(A)** HL-60, **(B)** THP-1, and **(C)** MV4-11 cells. **(D)-(I)** Representative graphs demonstrated the flow cytometric analysis of Na_2EA-induced cellular apoptosis. **(D)** and **(E)** HL-60 cells, **(F)** and **(G)** THP-1 cells, and **(H)** and **(I)** MV4-11 cells.

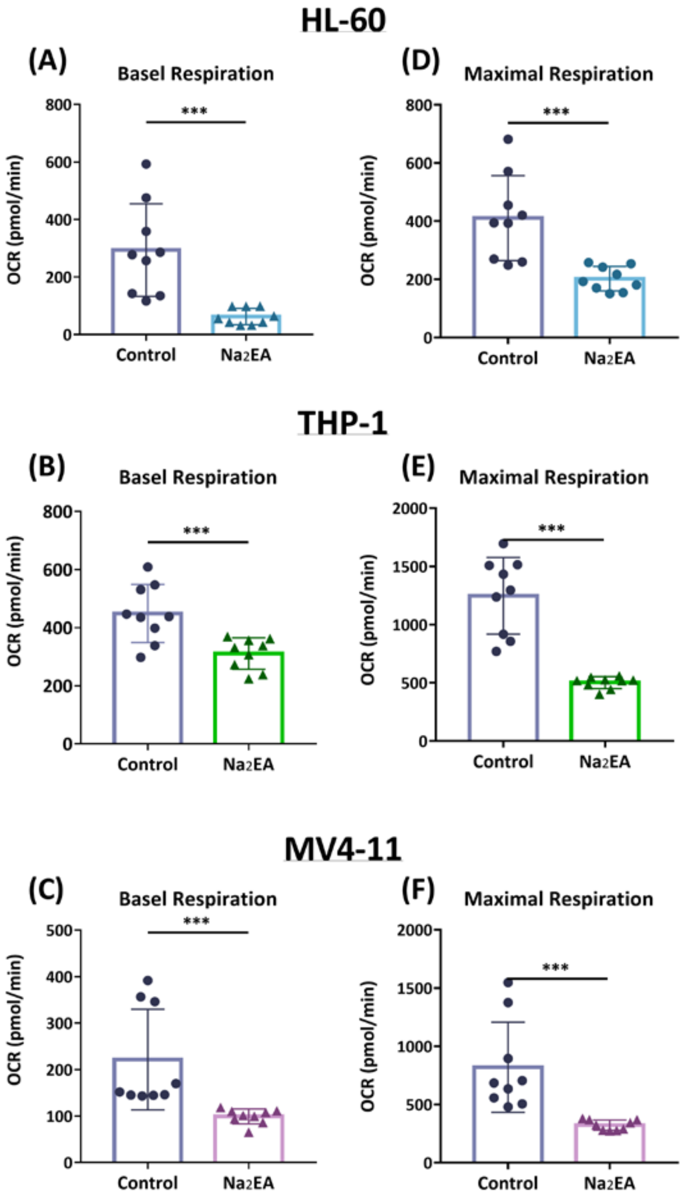

Figure S8. Basal and maximal respiration rates of Na_2EA-treated AML cells. (A), **(B)** and **(C)** The basal respiration of HL-60, THP-1, and MV4-11 cells, respectively. **(D)**, **(E)** and **(F)** The maximal respiration of HL-60, THP-1, and MV4-11 cells, respectively. The bar graphs illustrate the basal and maximal respiration rates (multiple numbers in each group, mean ± SD). *$p < 0.05$, **$p < 0.01$, and ***$p < 0.001$. p values were calculated using the Student's t-test.

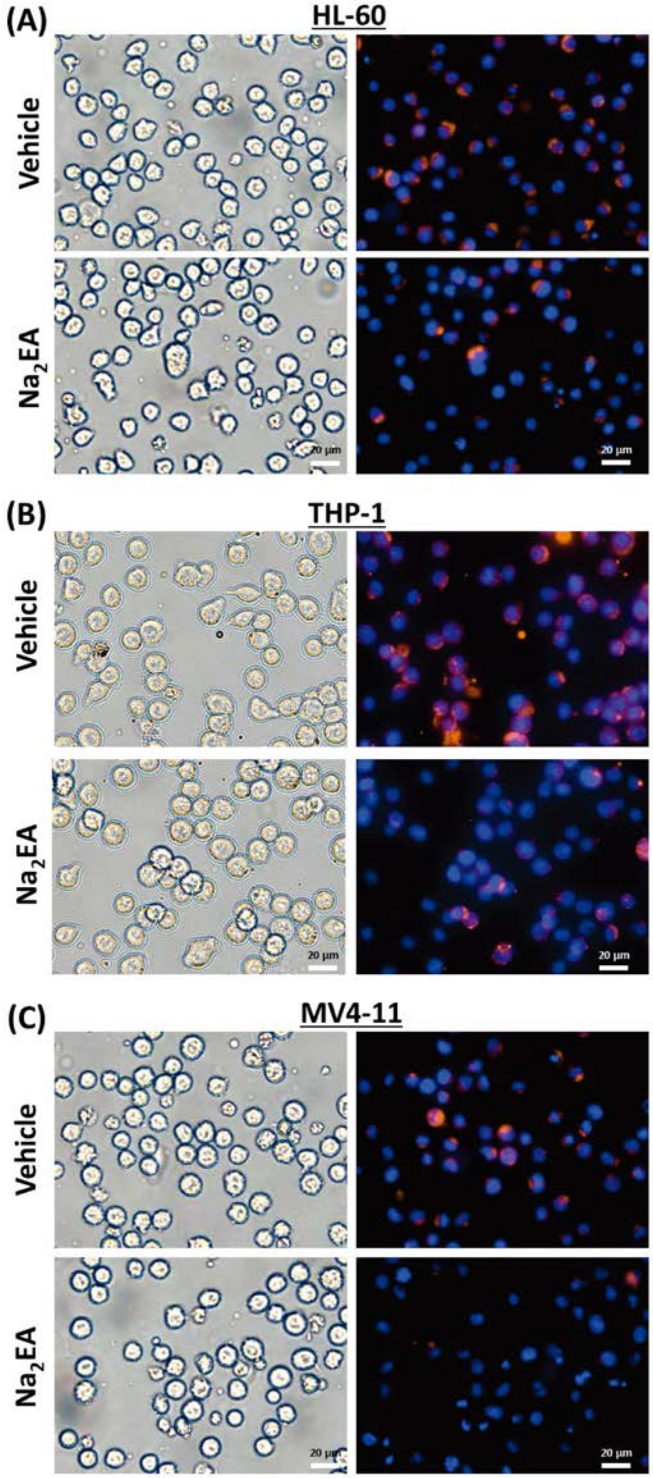

Figure S9. Living cell red fluorescent imaging for ATP. Representative graphs indicated the red fluorescence-labeled ATP in Na_2EA-treated **(A)** HL-60, **(B)** THP-1, and **(C)** MV4-11 cells.

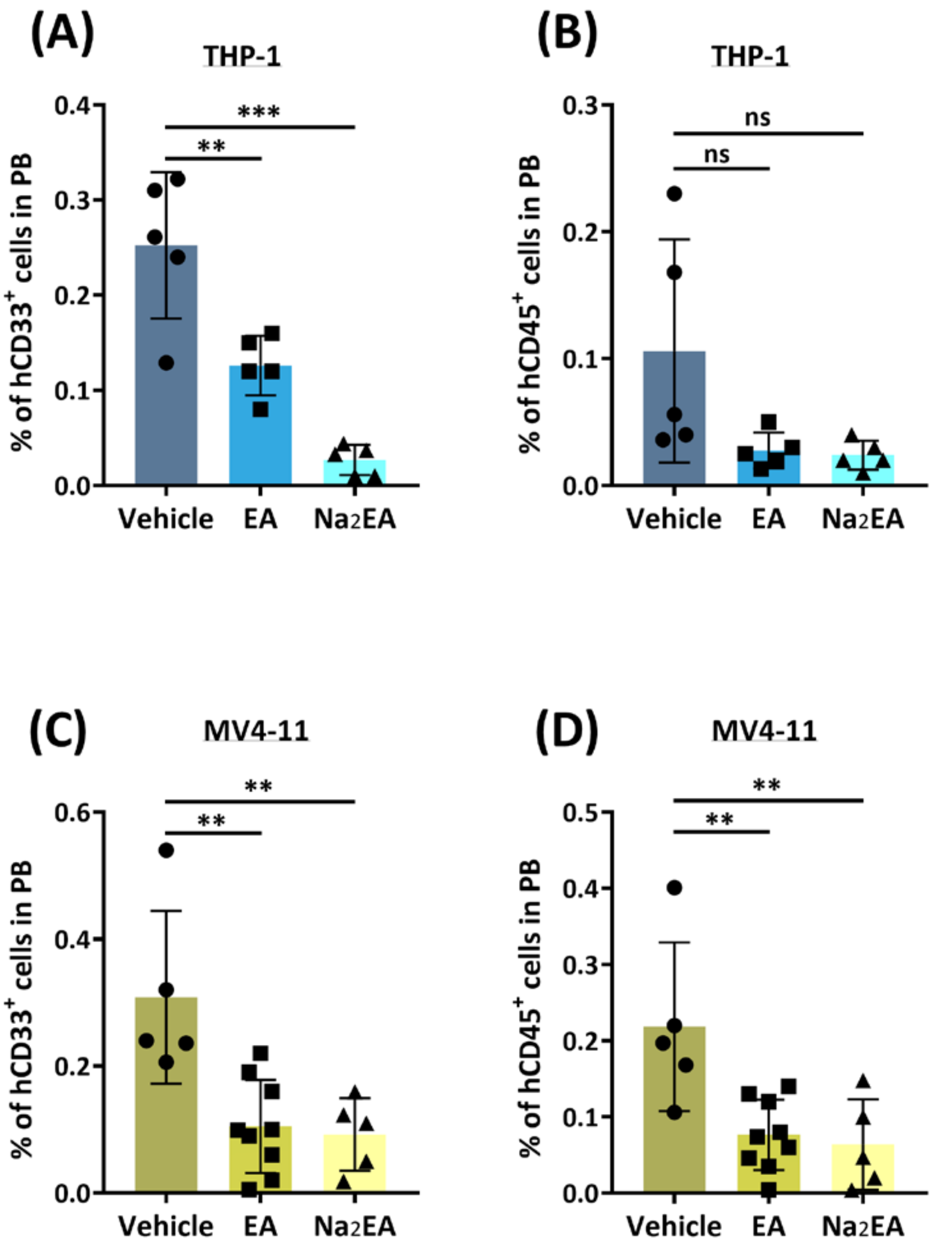

Figure S10. Antileukemic efficacy of ME2 inhibitors EA and Na_2EA in THP-1 and MV4-11-disseminated ASID mice after three weeks of xenotransplantation. **(A)** and **(B)** Percentage of CD33+ and CD45+ cells, respectively, in EA or Na_2EA-treated THP-1 cells in the peripheral blood (PB) of mice after three weeks. **(C)** and **(D)** Percentage of CD33+ and CD45+ cells, respectively, in EA or Na_2EA-treated MV4-11 cells in the peripheral blood (PB) of mice after three weeks.

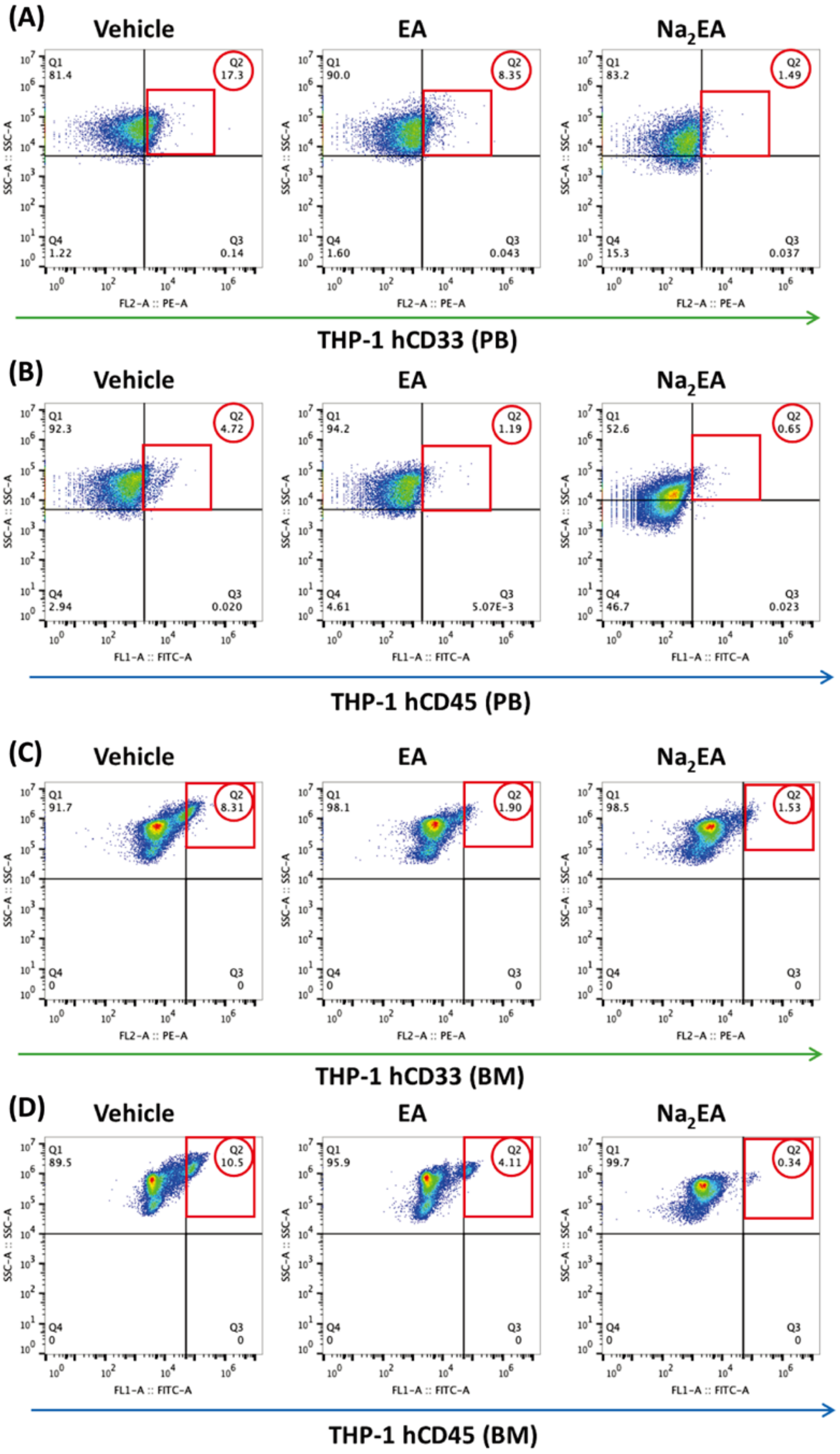

Figure S11. Representative graphs of the flow cytometric analysis of human CD33+ or CD45+ cells from ASID mice with EA or Na_2EA-treated THP-1 cells. (A) and **(B)** Flow cytometric analysis of the

CD33+ and CD45+ cells, respectively, in EA or Na_2EA-treated THP-1 cells in the peripheral blood (PB) of mice. **(C)** and **(D)** Flow cytometric analysis of the CD33+ and CD45+ cells, respectively, in EA or Na_2EA-treated THP-1 cells in the bone marrow (BM) of mice.

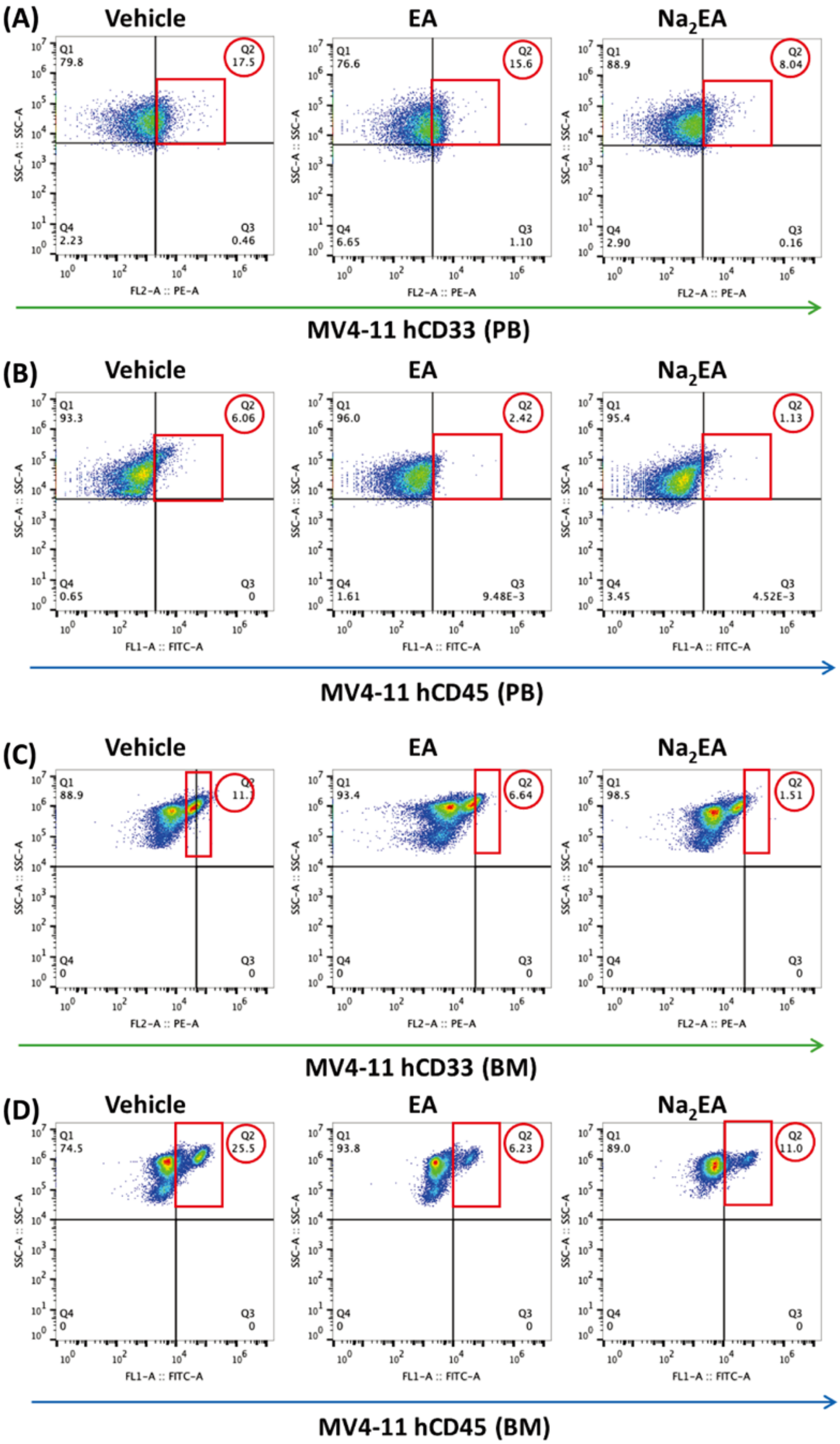

Figure S12. Representative graphs of the flow cytometric analysis of human CD33+ or CD45+ cells from ASID mice with EA or Na_2EA-treated MV4-11 cells. (A) and **(B)** Flow cytometric analysis of the

CD33+ and CD45+ cells, respectively, in EA or Na_2EA-treated MV4-11 cells in the peripheral blood (PB) of mice. **(C)** and **(D)** Flow cytometric analysis of the CD33+ and CD45+ cells, respectively, in EA or Na_2EA-treated MV4-11 cells in the bone marrow (BM) of mice.
